# Olfactory bulb anomalies in KBG syndrome mouse model and patients

**DOI:** 10.1186/s12916-024-03363-6

**Published:** 2024-04-15

**Authors:** Kara Goodkey, Anita Wischmeijer, Laurence Perrin, Adrianne E. S. Watson, Leenah Qureshi, Duccio Maria Cordelli, Francesco Toni, Maria Gnazzo, Francesco Benedicenti, Monique Elmaleh-Bergès, Karen J. Low, Anastassia Voronova

**Affiliations:** 1 Department of Medical Genetics, Faculty of Medicine & Dentistry, University of Alberta, Edmonton, AB T6G 2H7 Canada; 2 Women and Children’s Health Research Institute, University of Alberta, 5-083 Edmonton Clinic Health Academy, Edmonton, AB T6G 1C9 Canada; 3 Clinical Genetics Service and Coordination Center for Rare Diseases, Department of Pediatrics, Regional Hospital of Bolzano, Bolzano, Italy; 4 Clinical Genetics Unit, Hôpital Robert-Debré, Paris, France; 5 IRCCS Istituto Delle Scienze Neurologiche Di Bologna, UOC Neuropsichiatria Dell’età Pediatrica, Bologna, Italy; 6 IRCCS Istituto Delle Scienze Neurologiche Di Bologna, Programma Di Neuroradiologia Con Tecniche Ad Elevata Complessità (PNTEC), Bologna, Italy; 7 Laboratory of Medical Genetics, Translational Cytogenomics Research Unit, Bambino Gesù Children’s Hospital, IRCCS, 00165 Rome, Italy; 8 Service d’Imagerie Pédiatrique, Hôpital Universitaire Robert Debré, Paris, France; 9 Department of Academic Child Health, Bristol Medical School, Population Health Sciences, University of Bristol, Bristol, UK; 10 Clinical Genetics Service, St. Michaels Hospital, Bristol, UK; 11 Department of Cell Biology, Faculty of Medicine & Dentistry, University of Alberta, Edmonton, AB T6G 2H7 Canada; 12 Faculty of Medicine & Dentistry, Neuroscience and Mental Health Institute, Edmonton, AB T6G 2E1 Canada

**Keywords:** Anosmia, Olfactory nerve, Olfactory bulb, Rostral migratory stream, RMS, Neural stem cell, Epigenetics, Chromatin, Neuroblast, Migration, *ANKRD11*

## Abstract

*ANKRD11* (ankyrin repeat domain 11) is a chromatin regulator and the only gene associated with KBG syndrome, a rare neurodevelopmental disorder. We have previously shown that Ankrd11 regulates murine embryonic cortical neurogenesis. Here, we show a novel olfactory bulb phenotype in a KBG syndrome mouse model and two diagnosed patients. Conditional knockout of *Ankrd11* in murine embryonic neural stem cells leads to aberrant postnatal olfactory bulb development and reduced size due to reduction of the olfactory bulb granule cell layer. We further show that the rostral migratory stream has incomplete migration of neuroblasts, reduced cell proliferation as well as aberrant differentiation of neurons. This leads to reduced neuroblasts and neurons in the olfactory bulb granule cell layer. In vitro, *Ankrd11*-deficient neural stem cells from the postnatal subventricular zone display reduced migration, proliferation, and neurogenesis. Finally, we describe two clinically and molecularly confirmed KBG syndrome patients with anosmia and olfactory bulb and groove hypo-dysgenesis/agenesis. Our report provides evidence that Ankrd11 is a novel regulator of olfactory bulb development and neuroblast migration. Moreover, our study highlights a novel clinical sign of KBG syndrome linked to *ANKRD11* perturbations in mice and humans.

**Supplementary Information:**

The online version contains supplementary material available at 10.1186/s12916-024-03363-6.

## Introduction

The mammalian olfactory bulb (OB) is a twin ovoid structure located directly behind the nasal cavity and is part of the central nervous system (CNS). The OB is one of the few CNS structures known to undergo postnatal and adult neurogenesis, which plays an important role in OB growth and neuronal plasticity [1–3]. While the main function of the OB is olfaction [4], it is also involved in mood regulation [5]. Olfactory bulbectomy or OB neuronal ablation induces depressive-like behaviors in rodents [6–8]. Moreover, reduction or loss of olfaction is linked to behavioral changes in patients with major depressive disorder, bipolar disorder, and schizophrenia [9, 10].

The OB hosts a large diversity of cell types, including neuronal and glial cells of various origins and timepoints. Glutamatergic excitatory neurons are formed embryonically from the local neural stem and progenitor cells (NPCs) located in the OB ventricular zone [11–14]. GABAergic inhibitory neurons (interneurons), which include parvalbumin, calretinin, calbindin (CALB +), and neurogranin (NG +) cells, are generated during embryonic and/or postnatal development from the NPCs located in the subventricular zone (SVZ), which lines the lateral ventricles [2, 13, 15, 16]. Glial cells, including astrocytes (GFAP +) and oligodendroglia (PDGFRα +), have supporting functions in the OB. While some glial cells, such as oligodendrocyte progenitor cells (OPCs), arise embryonically from the medial ganglionic eminence and anterior entopeduncular area NPCs, the majority of proliferation and migration of glial cells occurs postnatally from the SVZ [2, 13].

The OB hosts several discrete neuronal layers, known as the granule cell layer (GCL), the mitral cell layer (MCL), the external plexiform layer (EPL), and the glomerular layer (GL) [17]. Starting with the deepest layer, the GCL primarily contains inhibitory interneurons [18]. Mitral/tufted (M/T) excitatory neuronal cell bodies are in the next layer above termed MCL, whereas their lateral dendrites are in the EPL, located above the MCL [19]. The most superficial neuronal layer is the GL, which is composed of spherical glomeruli, containing periglomerular cells, dendrites from excitatory M/T cells, and axons from olfactory sensory neurons (OSN) whose cell bodies are located in the olfactory nerve layer (ONL) of the nasal cavity [20, 21]. Progenitors and neuroblasts that arrive in the OB do so via tangential migration from the SVZ along the rostral migratory stream (RMS). Once neuroblasts reach the GCL, they migrate radially to populate the remaining layers [13]. These neuroblasts contribute to postnatal and adult OB neurogenesis [13].

While adult OB neurogenesis is well characterized throughout rodent life, adult OB neurogenesis in humans is debated. Several studies have provided evidence of adult human OB neurogenesis persisting throughout adulthood, while others have found a sharp decrease in human OB neurogenesis after childhood (reviewed in [3, 16]). Nevertheless, OB neurogenesis does occur postnatally in both the rodent and human brain development [22–24]. Migrating neuroblasts are ensheathed by astrocytes and guided by a variety of molecular and structural cues including chemotactic factors, blood vessels, extracellular matrix (ECM) components, and adhesion molecules [25–27]. This process contributes to OB growth and neuronal plasticity [2, 28].

OB development and maintenance are regulated transcriptionally and by sensory experiences. Odor deprivation decreases the number and maturation of postnatal newborn granule cells [29, 30], while odor training increases OB adult neurogenesis [31]. With regard to gene expression regulation, most research has focused on the role of transcription factors, such as homeobox and T-box genes, during OB development [17, 32, 33], while chromatin and epigenetic regulation has been understudied. What is known is that chromatin regulators, such as HDAC2 (histone deacetylase 2), regulate adult OB neurogenesis via adult-born OB neuron survival and differentiation [34]. With regard to OB development, chromatin remodelers, such as MeCP2 (methyl-CpG binding protein 2; Rett syndrome disease gene), regulate OSN development in the olfactory epithelium [35, 36]. Moreover, HDACs regulate neurotransmitter expression in the OB interneurons [37].

ANKRD11 is a chromatin regulator that interacts with HDACs and HATs (histone acetylases) to regulate global gene expression [38–40]. *ANKRD11* is one of the most frequently de novo mutated genes in monogenic neurodevelopmental disorders (NDDs) and is moderately de novo mutated in autism spectrum disorder (ASD) [41–44]. Inherited or de novo mutations in or deletions of *ANKRD11* are associated with KBG syndrome (OMIM #148050; named after the initials of the first three patients) [41]. Patients with KBG syndrome can present with short stature, distinctive facial features, macrodontia, skeletal anomalies, congenital heart defects, intellectual disability, and seizures [45–49]. Many patients will also have one or a combination of neurobehavioral issues, such as attention-deficit/hyperactivity disorder (ADHD), hyperactive/restless behaviors, performance anxiety, low frustration tolerance, obsessive–compulsive, and impulsive-aggressive behaviors, ASD, and mood disorders such as depression [50–52]. CNS anomalies include dilated brain ventricles, heterotopias, callosal abnormalities, and seizures [45, 50, 53–56]. We have previously shown that Ankrd11 regulates murine NPC proliferation, differentiation, and neuron localization in the developing cortex and during adult OB neurogenesis [38]. Whether Ankrd11 regulates embryonic and/or postnatal OB development has not been previously studied.

Here, we show that the loss of *Ankrd11* in the embryonic murine NPCs in vivo leads to hypoplastic postnatal OB with reduced GCL layer. We further show that loss of Ankrd11 leads to reduced NPC proliferation, migration, and aberrant neurogenesis in vitro and in vivo. Clinical observations and magnetic resonance imaging (MRI) from two patients with molecularly confirmed KBG syndrome show absence or hypoplasia of olfactory nerves and bulbs and/or clinical anosmia. Together, our results identify a new role for Ankrd11 in OB development and a novel OB-related clinical phenotype linked to KBG syndrome.

## Materials and methods

### Ethical compliance

This study is in accordance with the Helsinki declaration and its following modifications. The reported KBG syndrome patients’ data were obtained through routine clinical care and are not considered research at the involved institutions; formal review board approval was therefore not requested. Patient 1 was initially evaluated and treated at the Rare Disease Unit and the Pediatric Neurology Unit of University Hospital S.Orsola-Malpighi—Bologna, and subsequently at the Clinical Genetics Unit, Regional Hospital Bolzano, Italy. Patient 2 was diagnosed and in follow-up at the Clinical Genetics Unit, Hôpital Robert-Debré, Paris, France. Both parents of patients 1 and 2, and patient 1 himself, provided written informed consent for genetic testing, and publication of clinical data and/or pictures, and they authorized the processing of personal data according to the Italian and French bioethics laws.

All animal use was approved by the Animal Care Committee of the University of Alberta in accordance with the Canadian Council of Animal Care policies.

### Patients

Patient 1 (see Fig. 7A–D) is a 20-year-old male, the only child of healthy non-consanguineous Caucasian parents. The boy was born at term (40 WG + 1 day) without complications. He did not present with feeding difficulties, but he consistently refuses to eat any kind of fruit or raw vegetables. At 10 years, his linear and weight growth parameters were mostly 40th–50th centile (clearly below parental target of > 90th centile), with a slightly smaller but normal head circumference. At the age of 11.5 years, he was first investigated because of excess weight gain without linear growth. Initially, a constitutional delay of puberty was suspected since his bone age was delayed. He was already known to be anosmic and at age 13, a deficit of growth hormone (GH) was diagnosed, in the absence of other endocrinological anomalies. He started biosynthetic GH therapy at the age of 13 years and 10 months when his height was 148.8 cm (18th centile, target height 188 cm), and shortly thereafter, normal pubertal development followed. GH therapy was stopped after 3 years, due to a lack of gain in length, and other health problems. His final height aged 20 is 168.5 cm (13th centile), his weight is 64 kg (27th centile), and his head circumference is 54.5 cm (34th centile). At the age of 16 years and 8 months, he presented with focal, secondary generalized epileptic seizures. Seizures were resistant despite treatment with valproic acid, levetiracetam, carbamazepine, perampanel, and clobazam. He developed a possible drug-induced liver injury 2 weeks after the introduction of low dose carbamazepine. Ongoing therapy with lamotrigine has been successful in seizure remission. The clinical diagnosis of KBG syndrome was molecularly confirmed by the identification of the de novo heterozygous variant c. 3221_3222delAA, p. Lys1074ArgfsTer27 in the *ANKRD11* gene. This loss-of-function mutation was previously reported in another patient with KBG syndrome [57]. Patient 1 developed typical KBG craniofacial dysmorphic features from puberty, such as a triangular face, heavy eyebrows, bulbous nose, full lips, large central incisors, and low anterior hairline. He also presents with thorax asymmetry, thoracal dextro-convex scoliosis, thoracal kyphosis, anteverted shoulders, hyperextensible knees and interphalangeal articulations of fingers, myopia, and corneal dystrophy of the right eye. Cardiac and renal ultrasound were normal. Audiometric testing remains within normal limits.

Psychomotor development of this patient was unremarkable; he was able to walk independently at 13 months and pronounced his first words at 10 months, followed by a fast progression of language development. His school performance was normal, with above-average results; however, he showed poor adaptive behavior and occasional tantrums. At the age of almost 13 years, because of suspicion of dysgraphia, a neuropsychological evaluation was performed, which concluded: normal cognitive profile in average-high range (WISC-IV total QI 119), motor coordination disorder particularly regarding visual-motor integration (ICD-10 F82 specific developmental disorder of motor function), and leading to dysgraphia (ICD-10 F81.8) at school, fragile emotional control, and poor adaptation to changes. His behavioral issues worsened over time: currently, he displays impulsive-aggressive and obsessive–compulsive behaviors, a reduced ability to relate to others and low self-confidence. He tends to be anxious and depressive and is currently unable to live or work independently. Mood and behavior might be negatively influenced by his at least partial awareness of his limitations. He is receiving psychotherapy. A recent neuropsychologic re-evaluation confirmed normal intellectual functioning profile, but with disharmony in the functions of motor integration, social, and relational correspondence, which still require specialist investigations.

Patient 2 (see Fig. 7I) is an 8-year-old girl born to French non-consanguineous parents. She came to genetic counseling for psychomotor retardation and olfactory bulb agenesis. An array and testing for fragile X syndrome were performed but they were both negative. The sequencing of the *EHTM1* gene was normal. An ID gene panel found a variant c.6152dupC de novo in *ANKRD11*. She has hypertelorism, plagiocephaly, upwardly sloping labial commissures, large incisors, and short fingers especially the fifth. The proband was described as a very calm baby. The neonatal period was marked by failure to thrive and gastric reflux which needed treatment. She sat around 14 months and walked alone at the age of 2 years and 6 months. Her first words appeared around 1 year of age. At the age of 8 years, she still has a severe language delay. She has behavioral disorders with frustration intolerance, emotional immaturity, and crises of anger. She has poor safety awareness and has concentration difficulties. She had recurrent ear nose and throat infections and recurrent left lumbar tumefaction of unknown etiology which can evoke a vascular malformation. Her height has followed − 1SD trajectory and her head circumference plots on 2SD.

### MRI

MRI exams were performed at the age of 14 years for patient #1 and 3 years for patient #2 with a 3 T (Tesla) scanner (MAGNETOM Skyra, Siemens Healthcare, Erlangen, Germany) equipped with a 64-channel head coil. High-resolution heavily T2-weighted sequences sagittal sequences were acquired, completed with coronal multiplanar reconstruction (MPR). Axial brain images were made in FLAIR T2-weighted, coronal brain images in FSE T2-weighted. For the nasoethomodial region sagittal and coronal multiplanar reformatting three-dimensional high resolution heavy T2-weighted images were made. Controls with normal appearing OB anatomy underwent the same MRI exam and the resulting images were generated as described above.

### Prevalence patient survey

We collaborated with the KBG Foundation to obtain a snapshot of parent or self-reported anosmia or hyposmia. Families who are members of the KBG Foundation Facebook group were asked to answer a Facebook poll and select from the options (1) no sense of smell; (2) reduced sense of smell; (3) normal sense of smell/never noticed a problem; or (4) not possible to know. We specified that change of sense of smell following COVID infection should not be reported in this poll.

### Animals

All mice were housed in a University of Alberta Animal Facility and serviced by Health Sciences Laboratory Animal Services (HSLAS). Mice were maintained on a 12-h light or dark cycle, and food and water were provided ad libitum. Embryos at developmental ages embryonic day (E)18.5 and postnatal mice at age postnatal day (P)5, 10, 15, and P30 of either sex were used for all experiments.

Ankrd11^fl/fl^ mice in which exon 7 of the *Ankrd11* gene is flanked by LoxP sites are described in [58, 59]. RosaYFP^STOP/STOP^ (B6.129X1-Gt(ROSA)26Sortm1(EYFP)Cos/J, stock # 006148) mice were obtained from Jackson Laboratories [60]. NestinCre^ERT2^ mice were obtained from [61] and used to induce recombination in Nestin + NPCs [62, 63]. To generate Ankrd11 NPC-specific conditional knockout mice, Ankrd11^fl/fl^;RosaYFP^STOP/STOP^ females were mated with Ankrd11^fl/fl^;RosaYFP^STOP/STOP^;NestinCre^ERT2^ male mice to generate Ankrd11^fl/fl^;RosaYFP^STOP/STOP^ (Ankrd11^Contol^) and Ankrd11^fl/fl^;RosaYFP^STOP/STOP^;NestinCre^ERT2^ (Ankrd11^nscKO^) progeny. Mice were mated in the evenings and a positive plug, determined in the morning, was considered as E0.5.

CD1 timed pregnant females were purchased from Charles River and used to foster Ankrd11^Contol^ and Ankrd11^nscKO^ pups as per previously published cesarean section and cross-fostering protocols [64, 65].

### Tamoxifen and BrdU injections

Pregnant murine mothers were injected once intraperitoneally (IP) with 90 mg/kg of tamoxifen (TMX; Sigma) at E14.5. TMX dosage was optimized using Ankrd11^fl/fl^ dams between P70 and P90 to maximize survival of pups while maintaining efficient recombination (data not shown). TMX was dissolved in a 9.5% ethanol in sunflower seed oil (Sigma) as described in [62, 63] to induce recombination in Nestin + NPCs. P14 pups were IP injected with 100 mg/kg dose of BrdU (Bromodeoxyuridine) dissolved in sterile 1 × PBS (phosphate buffered saline) 24 h prior to euthanasia [66].

### Genotyping

All mice were bred and genotyped as recommended by Jackson Laboratories and European Mouse Mutant Archive (EMMA) using the following primers: Ankrd11: forward, 5’-CTGTCTCAGAGAGGAGAGTGAGGAGGAC-3’; reverse, 3’-TACCTTACACCCTGAGACGGCGTC-5’, 34 cycles of: 94 °C-30 s, 62 °C-45 s, 72 °C-60 s. Pan-Cre: forward, 5’-TTCCCGCAGAACCTGAAGATG-3’; reverse, 3’-11 CCCCAGAAATGCCAGATTACG-5’; control forward primer 5’-AACAACAATGGCACAACCTAAT-3’; control reverse 3’-ACTTTCTCCCCACCCGTCTA-5’; 35 cycles of: 94 °C-15 s, 60 °C-30 s, 72 °C-90 s. YFP^STOP^: wild-type, 5’-GGAGCGGGAGAAATGGATATG-3’; common, 5’-AAAGTCGCTCTGAGTTGTTAT-3’; mutant, 5’-AAGACCGCGAAGAGTTTGTC-3’, 35 cycles of 94 °C-15 s, 65 °C-15 s, 72 °C-60 s.

### Primary cultures

#### Primary neurospheres

Cells from embryonic cortex or postnatal SVZ were mechanically triturated and cultured at clonal density (10 cells/μl) as described in [67]. Briefly, cells were cultured in a 6-well polystyrene microplate (08–772-1B; Fisher) for 6 days in vitro (DIV) in 2 ml/well of serum-free media (SFM: equal parts Dulbecco’s modified Eagle medium low glucose (DMEM, 10567–014, (Sigma), 5 mM HEPES (Gibco), 0.1125% sodium bicarbonate (NaHCO3, Gibco), 1% penicillin–streptomycin (Gibco), and 100 μg/mL L-glutamine (Gibco), supplemented with 10 ng/mL FGF (fibroblast growth factor; Peprotech), 2% B27 (Gibco), 20 ng/mL EGF (epidermal growth factor; Peprotech), and 2 μg/mL heparin sodium salt (H3149-50KU; Sigma) [38, 67, 68].

For E15.5 cultures, TMX was injected into pregnant murine mothers at E14.5 as described above, and the cerebral cortex was microdissected from E15.5 Ankrd11^control^ and Ankrd11^nscKO^ embryos to generate primary neurospheres. Primary neurospheres were then centrifuged at 465* g* for 7 min at room temperature and the cell pellet was collected for total RNA purification according to manufacturer’s instructions (Omega Bio-Tek E.Z.N.A kit).

For P7 cultures, the SVZ was dissected from naïve (non-injected) Ankrd11^fl/fl^;RosaYFP^STOP/STOP^ and Ankrd11^fl/fl^;RosaYFP^STOP/STOP^;NestinCre^ERT2^ pups. Cells were cultured in a 6-well polystyrene microplate (08–772-1B; Fisher) with 2 ml of supplemented SFM per well for 6 DIV as described in [67]. To induce recombination in vitro, primary neurospheres on 5 DIV were incubated with 1 μM 4-hydroxytamoxifen (4OH-TMX) for 24 h. Primary neurospheres on 6 DIV were used in downstream assays described below.

#### Secondary neurospheres

P7 4-OHT treated primary neurospheres were pooled and dissociated as in [67]. Resulting neurosphere cells were cultured at clonal density (2 cells/μl) as secondary neurospheres as described in [67] for 7 DIV in a 6-well polystyrene microplate with 2 ml of SFM supplemented with FGF, EGF, B27, and heparin. Secondary neurospheres were counted and then centrifuged at 465* g* for 7 min at room temperature, and the cell pellet was collected for total RNA purification according to manufacturer’s instructions (Omega Bio-Tek E.Z.N.A kit).

#### Primary neurosphere migration assay

Ten to 15 of P7 4-OHT treated primary neurospheres were randomly selected and plated on a 40 μg/mL poly-D-lysine (PDL) (Sigma, P6407) and 4 μg/mL laminin (VWR, CACB354232) coated coverslips [69, 70]. Spheres were allowed to adhere to the plate for 5–10 min in the incubator prior to the addition of SFM supplemented with FGF, EGF, and B27 [67]. Individual spheres were imaged at plating and 24 h later (1 DIV).

#### NPC scratch assay

P7 4-OHT treated primary neurospheres were pooled and dissociated as in [67]. Resulting neurosphere cells were plated at high density (260,000 cells/cm^2^) on PDL- and laminin-coated 24-well plates in SFM supplemented with FGF, EGF, and B27. After 3–4 h (after NPCs adhered), a single scratch was made in each well using a 10-μl pipette tip. Images of each scratch were taken directly post scratch (0 DIV), 1, and 2 DIV.

#### NPC monolayer cultures

P7 4-OHT treated primary neurospheres were pooled and dissociated as in [67]. Resulting neurosphere cells were plated at 40,000 cells/cm^2^ density, optimal for differentiation into neurons, astrocytes, and oligodendroglial cells [67], on PDL- and laminin-coated coverslips. Cells were cultured in SFM supplemented with FGF, EGF, and B27 for 4 DIV [67].

### qRT-PCR (quantitative reverse transcriptase-polymerase chain reaction)

cDNA was generated from 20 to 70 ng of total RNA isolated from primary or secondary neurospheres using QuantiTect Reverse Transcription Kit (Qiagen) as per the manufacturer’s instructions. A no-RT control was included to verify genomic DNA elimination. 1/100th of the RT reaction was used as a template for the QPCR amplification. QPCR was performed according to MIQE guidelines [71] using the Ssoadvanced SYBR Green kit (BioRad) on the Roche LightCycler 96 machine with the following primers: *Ankrd11* exon 7: Forward 5’-GCGTGTAACCGGGGCTATTAC-3’; Reverse 5’-CGTTGTTGGCGGCATCATG-3’, 40 cycles of; 95 °C 15 s, 60 °C 15 s, 72 °C 20 s. *Gapdh*; Forward 5’-AAATACGGACTGCAGCCCTC-3’; Reverse 5’-AAATCCGTTCACACCGACCTT-3’, 40 cycles of; 95 °C 15 s, 60 °C 15 s, 72 °C 20 s.

### Immunocytochemistry and Immunohistochemistry

#### Tissue preparation

**Fig. 1:**
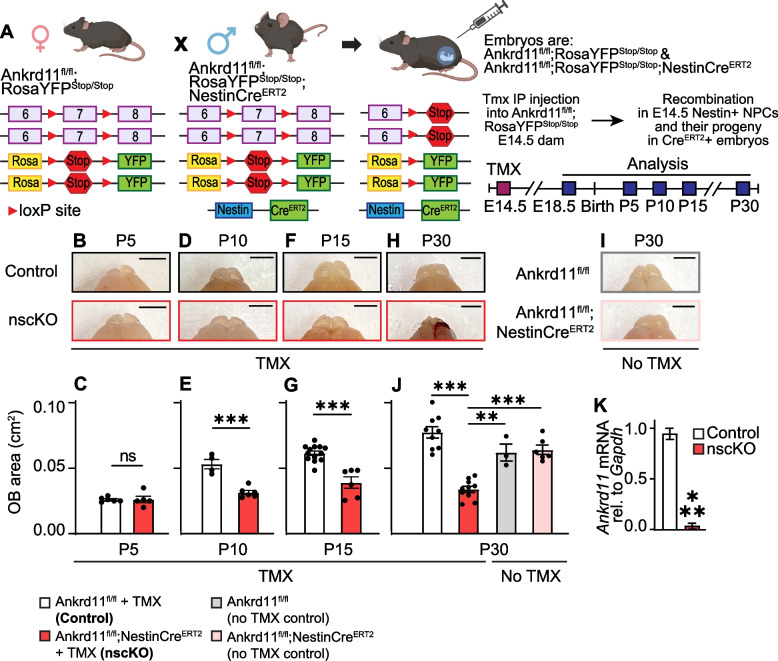
Gross morphological changes in postnatal Ankrd11^nscKO^ OB. **A** Schematic of breeding scheme. Ankrd11^fl/fl^;RosaYFP^STOP/STOP^ females were mated with Ankrd11^fl/fl^;RosaYFP^STOP/STOP^;NestinCre^ERT2^ males. Pregnant dams were injected with tamoxifen (TMX) at E14.5. Resulting embryos (Ankrd11^control^ and Ankrd11^nscKO^) were collected at E18.5 or allowed to be born with subsequent collection at P5-P30. Loss of *Ankrd11* only occurred after TMX injection in embryos carrying NestinCre^ERT2^ allele. **B–I** Representative images of Ankrd11^control^ and Ankrd11^nscKO^ OB at P5 (**B**), P10 (**D**), P15 (**F**), and P30 (**H**) that were injected with TMX at E14.5. Uninjected Ankrd11^fl/fl^ and Ankrd11^fl/fl^;NestinCre^ERT2^ control images have gray and pink borders in (**I**). Quantification of OB area of Ankrd11^control^ and Ankrd11^nscKO^ at P5 (**C**), P10 (**E**), P15 (**G**), and P30 (**J**). Gray and pink bars in **J** show P30 OB area from uninjected Ankrd11^fl/fl^ and Ankrd11^fl/fl^;NestinCre^ERT2^. Scale bar = 250 mm. **K** Quantitative RT-PCR for *Ankrd11* mRNA in E15.5 cortical primary neurosphere cultures dissected from Ankrd11^control^ and Ankrd11^nscKO^ embryos that received TMX at E14.5. Data were normalized to *Gapdh* mRNA and calibrated against Ankrd11^Control^ samples. Error bars represent SEM. *n* = 5–13 mice per genotype from at least 2 independent litters. Data were analyzed using unpaired *t*-test (**C**,**E**,**G**,**K**) or two-way ANOVA followed by Tukey’s multiple comparisons post hoc test (**J**), ns not significant, **p < 0.01, ***p < 0.001. Ctrl control, E embryonic day, P postnatal day, TMX tamoxifen, YFP yellow fluorescent protein, OB olfactory bulb

Pregnant murine dams were euthanized with CO2 and brains from E18.5 embryos were dissected and incubated in 4% paraformaldehyde (PFA, Fisher, 50980487) for 24 h at 4 °C. P15 pups were euthanized via IP injection of Euthanyl (Bimeda-MTC) at 192 mg/kg of body weight, followed by transcardiac perfusion with HBSS (Hanks’ Balanced Salt Solution, Gibco) and 4% PFA. At this point, postnatal brain areas encompassing OB were photographed using an iPhone 11 pro (Apple) at 2 × zoom to generate top-down images in Fig. 1B–I. Dissected brains were further fixed in 4% paraformaldehyde for 24 h at 4 °C, after which tissues were incubated in 30% sucrose in 1 × PBS for 72 h. Samples were embedded and flash frozen in an optimal cutting temperature (OCT) compound (Epredia) and sectioned sagitally with 16 µm thickness on a cryotome (Leica Biosystems, Germany). The sections were transferred onto glass microscope slides and stored at − 80 °C.

#### Immunofluorescence

Sections were processed for immunohistochemistry as described in [62, 63, 66]. Briefly, sections were rehydrated with PBS and blocked for 1 h with 5% bovine serum albumin (BSA, Jackson ImmunoResearch) and 0.3% Triton-X100 in PBS, then incubated with primary antibodies listed in the “Antibodies” section diluted in 5% BSA overnight at 4 °C. Sections were then washed in PBS and incubated with appropriate secondary antibodies listed in the “Antibodies” section for 1 h at room temperature. Some sections also underwent BrdU (Bromodeoxyuridine) staining, which was performed as described in [63]. Briefly, immunostained sections were post-fixed with 4% PFA and underwent bathing in 1 M and 2 M HCL, followed by incubation with anti-BrdU primary antibody and appropriate secondary antibody (see “Antibodies” section below) incubation thereafter. Samples were then counterstained with the nuclear stain Hoechst (33217; Riodel-De Haen Ag) at 1:1000, washed extensively with PBS, and mounted with Fluoromount-G mounting media (ThermoFisher). Whenever possible, immunostaining was performed on all slides at once to limit batch-to-batch variability.

Four DIV NPC monolayer cultures were immunostained as described in [62]. Briefly, cultures were fixed with 4% PFA, washed in PBS, permeabilized with 0.2% IGEPAL CA-630 (Sigma) solution, and blocked with 6% donkey serum and 0.5% BSA in PBS for 1 h. Fixed cultures were then incubated with primary antibodies overnight at 4 °C and appropriate secondary antibodies for 1 h at room temperature (see “Antibodies” section below for more details). Samples were counterstained with Hoechst as above and mounted onto glass coverslips with Fluoromount-G mounting media (ThermoFisher).

### Antibodies

Primary antibodies: anti-BrdU (Abcam, ab1893, RRID:AB_302659, 1:1000), anti-βIII-tubulin (Biolegend, 802001, RRID:AB_2564645, 1:2000), anti-CALB (Swant, cb38, RRID:AB_10000340, 1:1000), anti-CC3 (Millipore, AB3623, RRID:AB_91556, 1:250), anti-DCX (Abcam, ab18723, RRID:AB_732011, 1:300), anti-GFAP (Thermo Fisher, 13–0300, RRID:AB_2532994, 1:2000), anti-NEUN-Alexa555 (Millipore, MAB377A5, RRID:AB_2814948, 1:500), anti-NG (Abcam, AB5620, RRID:AB_91937, 1:600), anti-PSA-NCAM (Sigma, MAB5324, RRID:AB_95211, 1:400), anti-PDGFRα (R&D Systems, AF1062, RRID:AB_2236897, 1:400), and anti-SOX2 (Novus Bio, AF2018, RRID:AB_355110, 1:1000 or Cell Signaling, 3728S, RRID:AB_2194037, 1:1500).

Secondary antibodies: anti-goat-555 (Jackson, 705–165-147, RRID:AB_2307351, 1:1000), anti-goat-647 (Jackson, 705–605-147, RRID:AB_2340437, 1:1000), anti-mouse IgM-555 (Jackson, 715–165-140, RRID:AB_2340812, 1:1000), anti-rabbit-488 (Jackson, 711–545-152, RRID:AB_2313584, 1:1000), anti-rat-555 (Jackson, 712–165-153, RRID:AB_2340667, 1:1000), and anti-sheep-647 (Jackson, 713–175-147, RRID:AB_2340730, 1:1000).

### Microscopy

Live images of neurospheres as well as NPC scratch assay were captured with a Zeiss Primovert inverted cell culture microscope, Zeiss Axiocam ERc5s camera, and Zen software (Zeiss). Primary neurospheres or NPCs that underwent scratch assays (migration assays) were imaged at 100 × magnification. Secondary neurospheres were swirled into the center of the well and imaged at 40 × magnification.

Immunostained brain sections and cultures were imaged with a Zeiss Axio Imager M2 fluorescence microscope, ORCA-Flash LT sCMOS Camera, and Zen software (Zeiss). Four to six sections in every sample encompassing medial and lateral planes were imaged at 100 × magnification in a single plane. Representative images show tiled images encompassing OB in a sagittal plane or cropped images encompassing OB neuronal layers. Images from each set of immunostaining were acquired using identical settings.

### Data analysis

All data were analyzed and presented from at least three animals per genotype. E18.5 samples were collected from two independent litters and P15 samples were collected from three independent litters. Cell culture data was obtained from at least two independent litters. Sample sizes (*n*) are indicated in the figure legends and indicate the number of animals analyzed. All data were graphed using GraphPad Prism (version 8.0.2) and presented as mean ± SEM. Representative images were acquired with Zen software and processed in Adobe Photoshop CC 2015. Figures were generated in Adobe Illustrator CC 2015. Schematics were generated using Biorender and Adobe Illustrator.

#### qRT-PCR analysis

Quantitative RT-PCR data were collected from 13 E15.5 embryos (5 Ankrd11^nscKO^ and 8 Ankrd11^Control^) and 6 P7 pups (3 Ankrd11^nscKO^ and 3 Ankrd11^Control^) performed in technical duplicates. Average cycle threshold (Ct) values were normalized to *Gapdh* and calibrated against a Ankrd11^control^ sample from each litter using the 2^−ΔΔCt^ method [72].

#### Image analysis

Data analysis from E18.5 brains was blinded to the genotype until analysis completion. Since Ankrd11^nscKO^ postnatal mice had visibly smaller OBs, blinded analysis at this age was not possible. Data at least from eight animals from two independent litters was analyzed for all ages.

**Fig. 2:**
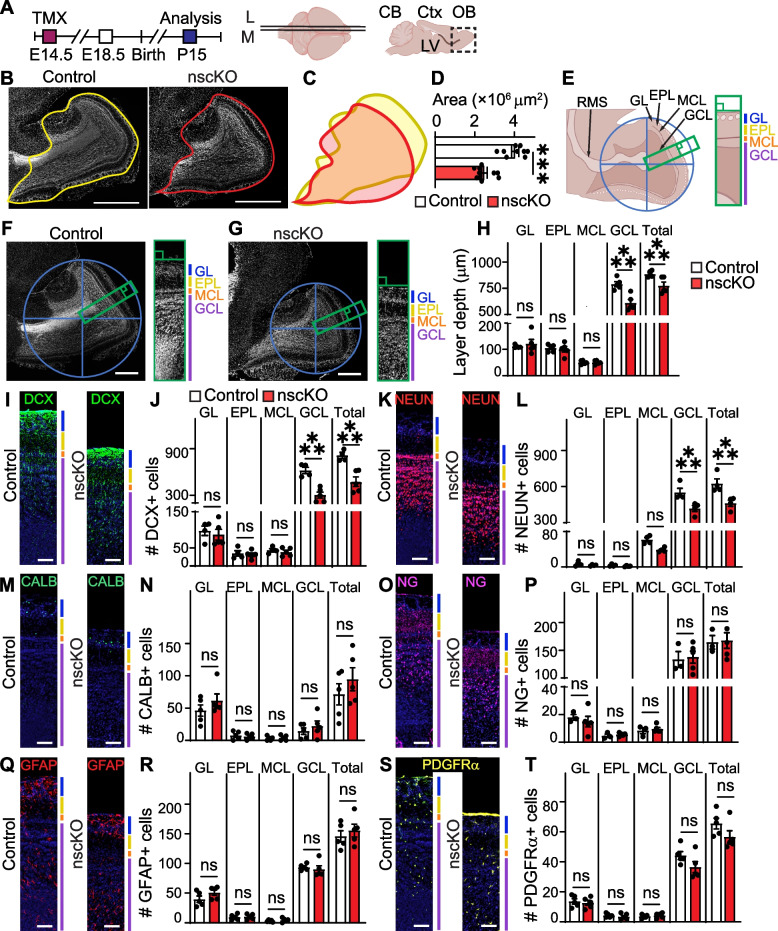
Loss of *Ankrd11* in E14.5 NPCs results in reduced P15 OB with aberrant cellular composition. Also see Supplemental Fig. 1*.* **A** Ankrd11^fl/fl^;RosaYFP^STOP/STOP^ dams mated with Ankrd11^fl/fl^;RosaYFP^STOP/STOP^;NestinCre^ERT2^ males were injected with TMX at E14.5. Resulting pups (Ankrd11^control^ and Ankrd11^nscKO^) were collected at P15. Images were collected from anatomically matched medial (M) and lateral (L) sagittal sections encompassing OB. **B** Representative images of Ankrd11^control^ and Ankrd11^nscKO^ sagittal sections stained with Hoechst (white). Solid outline shows OB area (excluding ONL) of Ankrd11^control^ (yellow) and Ankrd11^nscKO^ (red). **C** Representative overlap of Ankrd11^control^ and Ankrd11^nscKO^ OB area from (**B**). **D** Quantification of **B**. **E** Representative P15 OB schematic that displays identification of OB center by circle and crosshairs method followed by demarcation of the OB column and layers. Please see “Materials and methods” for more details. RMS rostral migratory stream, GL glomerular layer, MCL mitral cell layer, EPL external plexiform layer, GCL granule cell layer. **F**, **G** Representative images of Ankrd11^control^ (**F**) and Ankrd11^nscKO^ (**G**) OB stained with Hoechst (white), blue crosshair shows center of OB used to demarcate the OB column (green rectangle). OB column crop is on the right side with demarcated layers. **H** Quantification of OB layer depth shown in **F** and **G**.** I–T** Representative images and quantification of marker + cells in Ankrd11^control^ and Ankrd11^nscKO^ OB column layers immunostained for DCX (green) (**I**, **J**), NEUN (red) (**K**, **L**), CALB (green) (**M**, **N**), NG (pink) (**O**, **P**), GFAP (red) (**Q**, **R**), and PDGFRα (yellow) (**S**, **T**). Hoechst is counterstained in blue. The Hoechst panels in **I**, **Q**, and **S** were obtained from the same image immunostained for DCX, GFAP, and PDGFRα; and in **K** and **M** from the same image immunostained for NEUN and CALB. Error bars represent SEM. Data was analyzed using two-way ANOVA followed by Tukey’s multiple comparisons post hoc test. *n* = 5 mice per genotype from at least two independent litters. Scale bars represent 1000 μm (**B**), 500 μm (**F** and **G**), 100 μm (**I**, **K**, **M**, **O**, **Q** and **S**). CB cerebellum, Ctx cortex, E embryonic day, L lateral, M medial, OB olfactory bulb, P postnatal day

In Fig. 1, area of whole OBs was outlined in ImageJ and measured. In Figs. 2 and S1, area of one OB (excluding ONL) in anatomically matched sagittal sections was outlined in ImageJ and measured. GL and ONL were identified using Hoechst signal.

**Fig. 3:**
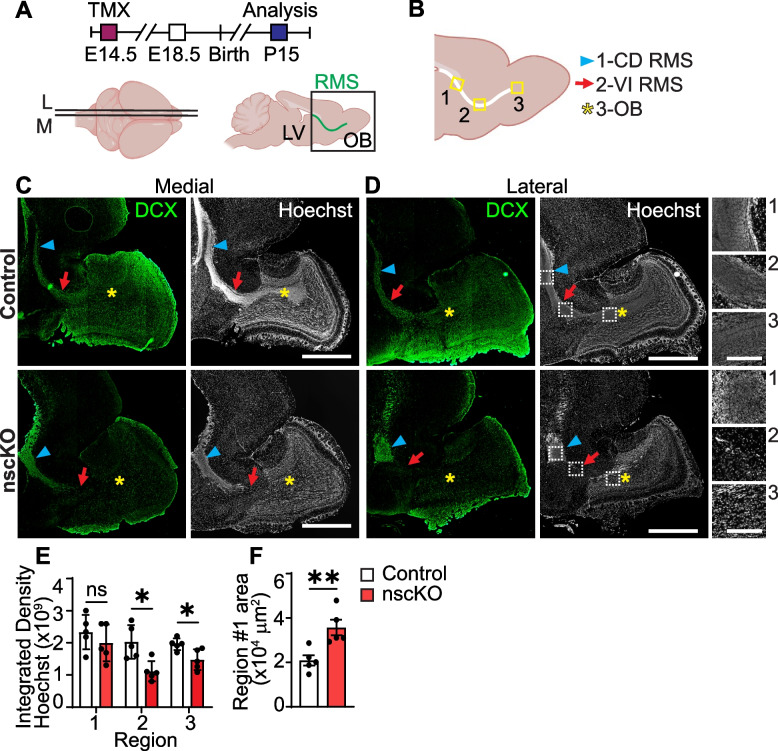
Loss of *Ankrd11* in E14.5 NPCs leads to abrogated P15 RMS migration. Also see Supplemental Fig. 2. **A** Ankrd11^fl/fl^;RosaYFP^STOP/STOP^ dams mated with Ankrd11^fl/fl^;RosaYFP^STOP/STOP^;NestinCre^ERT2^ males were injected with TMX at E14.5. Resulting mice (Ankrd11^control^ and Ankrd11^nscKO^) were collected at P15. Images were collected from anatomically matched medial (M) and lateral (L) sagittal sections encompassing OB. RMS indicated in green. **B** Schematic of areas measured in P15 RMS analysis. Region 1 encompassed the caudodorsal (CD) zone in the RMS (indicated by a blue arrowhead), region 2 the ventral intermediate zone in the RMS (indicated by a red arrow), and region 3 where the RMS meets the OB (indicated by a yellow star). **C**, **D** Representative images from Ankrd11^control^ and Ankrd11^nscKO^ P15 medial (**C**) and lateral (**D**) OB encompassing RMS immunostained for DCX (green) and Hoechst (white). Blue arrowhead indicates CD RMS (1), red arrow indicates VI RMS (2), yellow star indicates OB (3). White dashed boxes in lateral Hoechst panel in **D** are shown in the right column at higher magnification and labeled with regions #1–3 corresponding the regions identified in **B**. **E** Quantification of integrated density of Hoechst signal in CD RMS (1), VI RMS (2) and OB (3) regions outlined in **D**. **F** Quantification of region #1 (CD RMS) area. Error bars represent SEM. Data was analyzed using unpaired *t*-test. ns not significant, *p < 0.05, **p < 0.01, ***p < 0.001. *n* = 4–5 mice per genotype from at least two independent litters. Scale bars represent 1000 μm (**C, D**), 200 μm (insets of **D**). E embryonic day, L lateral, LV lateral ventricle, M medial, OB olfactory bulb, RMS rostral migratory stream

**Fig. 4:**
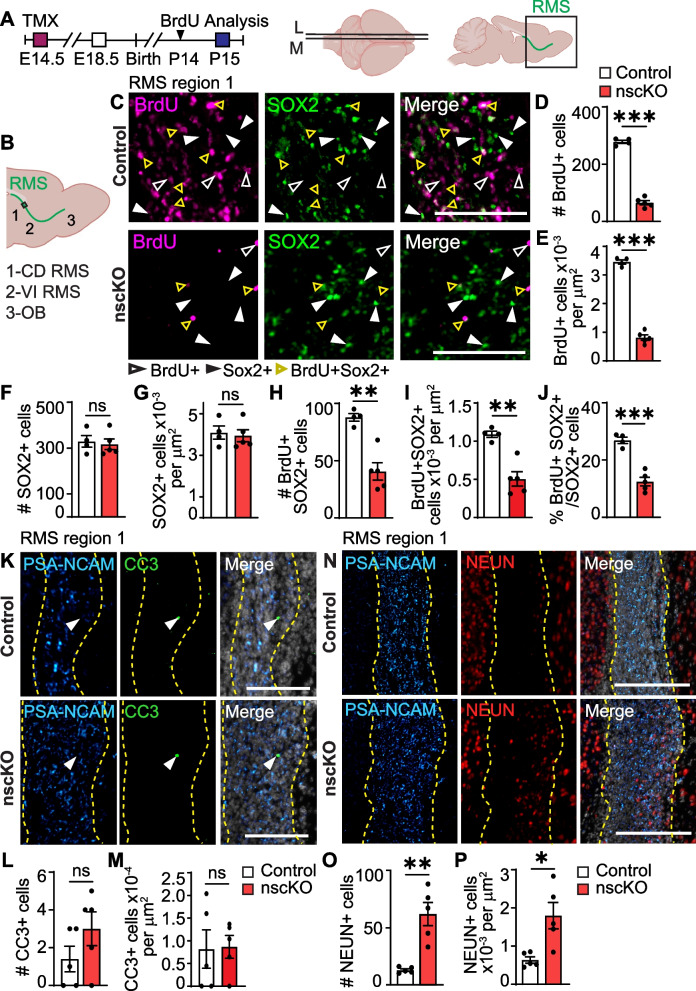
Loss of *Ankrd11* in E14.5 NPCs leads to decreased proliferation and aberrant neurogenesis in P15 RMS. Also see Supplemental Fig. 3*.* **A** Ankrd11^fl/fl^;RosaYFP^STOP/STOP^ dams mated with Ankrd11^fl/fl^;RosaYFP^STOP/STOP^;NestinCre^ERT2^ males were injected with TMX at E14.5 and resulting pups were injected with BrdU at P14. Resulting mice (Ankrd11^control^ and Ankrd11^nscKO^) were collected at P15. Images were collected from anatomically matched medial (M) and lateral (L) sagittal sections encompassing OB. RMS indicated in green. **B** Schematic of areas measured in P15 RMS analysis. Region 1 encompassed the caudodorsal (CD) zone in the RMS, region 2 the ventral intermediate zone in the RMS, and region 3 where the RMS meets the OB. Black box shows analysis area of region 1 in **C**. **C** Representative images of Ankrd11^control^ and Ankrd11^nscKO^ P15 RMS region 1 immunostained for BrdU (pink) and SOX2 (green). Open white arrow indicates BrdU + SOX2- cells, closed white arrow indicates BrdU-SOX2 + cells, and yellow open arrow indicates BrdU + SOX2 + cells. Please see Fig. S3A for approximate image locations. **D–J** Quantification of **C** for BrdU + cell number (**D**) and density (**E**), SOX2 + cell number (**F**) and density (**G**), BrdU + SOX2 + cell number (**H**) and density (**I**) and proliferative index of Sox2 + cells (% BrdU + SOX2 + /SOX2 + cells) (**J**). **K** Representative images of Ankrd11^control^ and Ankrd11^nscKO^ P15 RMS region 1 immunostained for PSA-NCAM (light blue) and CC3 (green). RMS outline in yellow dashed line is defined by the PSA-NCAM boundaries. White arrows indicate CC3 + cells. Counterstained with Hoechst in white. Please see Fig. S3C for approximate image locations. **L–M** Quantification of (**K**) for CC3 + cell number (**L**) and density (**M**). **N** Representative images of Ankrd11^control^ and Ankrd11^nscKO^ P15 RMS region 1 immunostained for PSA-NCAM (light blue) and NEUN (red). RMS outline in yellow dashed line is defined by the PSA-NCAM boundaries. Counterstained with Hoechst in white. PSA-NCAM image is also presented in Fig. S3C inset, along with a precise image location in the OB sections. **O–P** Quantification of (**N**) for NEUN + cell number (**O**) and density (**P**). Error bars represent SEM. Data was analyzed using unpaired *t*-test. ns not significant, *p < 0.05 **p < 0.01, ***p < 0.001. *n* = 4–5 mice per genotype from at least two independent litters. Scale bars represent 100 μm (**C, K**), 200 μm (**N**). E embryonic day, L lateral, LV lateral ventricle, M medial, OB olfactory bulb, P postnatal day, RMS rostral migratory stream

For analysis in Figs. 2, 3 and 4, S1-3, anatomically matched sections from each biological replicate were analyzed. Image contrast was enhanced to the same degree and only using linear enhancement. Due to the varying size of the sagittal OB area between Ankrd11^control^ and Ankrd11^nscKO^ animals, samples were anatomically matched by selecting the two largest OB areas from each animal that contained both visible RMS and well-defined OB layers. To identify the center of the OB in an unbiased manner, we overlayed a crosshair pattern over each anatomically matched OB section. We then applied a circle that would encompass OB borders. Finally, we applied the same size column (rectangle), which originated from the OB center and emanated outwards in a rostral direction (towards circle edge) (Figs. 2E, S1E). The edge of the column was aligned so it was perpendicular (green square indicating 90°) to the edge of the OB and yielded horizontally aligned OB layers for downstream analysis (Figs. 2F-G, S1F). Further identification of the OB neuronal layers was performed using Hoechst staining (Figs. 2, S1).

E18.5 and P15 OB sections were subjected to marker + cell counting as described in [38, 73]. Briefly, the entire OB was imaged and a defined size column spanning OB center to ONL was counted for marker + cells (Figs. 2E, S1E). Results are presented as mean marker + cells per entire column or per layer. For BrdU quantification, results are presented as mean number or density of BrdU + cells and as a proportion of BrdU + marker + cells relative to total marker + cells. Images in Figs. 3C, D, S2B and S3A were analyzed using integrated density, which integrates the area size with the mean gray value of the fluorescent signal, using Fiji software [74].

For RMS analysis, RMS was identified with PSA-NCAM or DCX immunostaining and divided into three regions; region 1 is a caudodorsal (CD) zone, region 2 is a ventral intermediate (VI) zone according to [75, 76], and region 3 is OB as depicted in Fig. 3B. Integrated density and area size for the three regions were analyzed as described above.

In all immunostained culture experiments, three to five random fields of view were captured with 20 × objective. For cell identity analysis, at least 300 cells from different fields were counted per condition and results are from at least two technical replicates from each biological replicate. Images were enhanced linearly and to the same extent to facilitate marker + cell analysis. Marker + cells are expressed as proportion of healthy (non-condensed) Hoechst + nuclei, except CC3 + cells are expressed as proportion of total (sum of cells with condensed and healthy) Hoechst + nuclei.

For secondary neurosphere analysis, each biological replicate contained 2–3 technical replicates and every sphere containing 50 or more cells was counted [63, 77]. Each data point in Fig. 6C represents an average number of spheres per technical replicate. After counting, spheres were swirled to the center and imaged as described above. Sphere diameter analysis was performed using ImageJ on spheres that fit within the field of view. In cases of uneven sphere shape, the longest diameter was recorded. A minimum of ~ 100 spheres per field of view was measured.

**Fig. 5:**
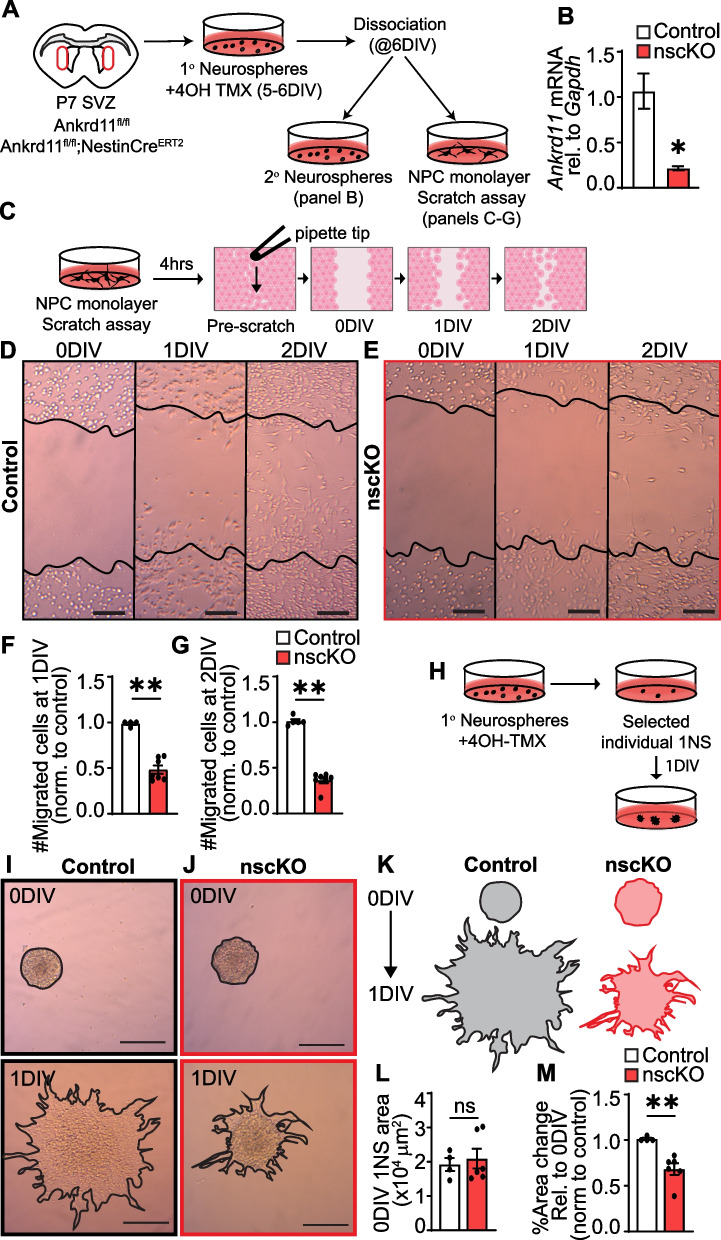
Loss of *Ankrd11* results in reduced SVZ NPC migration in vitro. **A** Schematic: P7 SVZ primary neurospheres were cultured from naive uninjected Ankrd11^fl/fl^ and Ankrd11^fl/fl^;NestinCre^ERT2^ pups for 6 DIV. Neurospheres were treated with 4OH-TMX for 24 h prior to dissociation on 6 DIV resulting in Ankrd11^Control^ and Ankrd11^nscKO^ NPCs. Treated primary neurospheres were directly plated onto adherent wells for primary neurosphere migration assay (**H–M**). Treated primary neurospheres were also dissociated and cultured as adherent NPC monolayer for scratch assay (**C–G**) or secondary neurospheres (**B**). **B** Quantitative RT-PCR for *Ankrd11* mRNA in P7 SVZ secondary neurosphere cultures from Ankrd11^control^ and Ankrd11^nscKO^ NPCs. Data were normalized to *Gapdh* mRNA and calibrated against Ankrd11^Control^ samples. **C** Schematic: 4OH-TMX treated P7 SVZ primary neurosphere cells were cultured as adherent NPCs and subjected to scratch assay. **D**, **E** Representative images of Ankrd11^control^ and Ankrd11^nscKO^ NPC scratch assay at 0, 1, and 2 DIV. 0 DIV indicates images taken immediately after scratch, and 1 and 2 DIV indicate images taken 1–2 days after scratch. Scratch region at 0 DIV is outlined in black. **F**, **G** Quantification of cell migration into scratch region at 1 DIV (**F**) and 2 DIV (**G**) normalized to control. **H** Schematic: 4 OH-TMX treated selected P7 SVZ primary neurosphere cells were allowed to adhere and neurosphere cell migration was assessed at 1 DIV. **I**, **J** Representative images of Ankrd11^control^ and Ankrd11^nscKO^ primary neurospheres assay at 0 and 1 DIV. Black outline shows the area of the neurosphere boundary (**K**). **L **Quantification of primary neurosphere area at 0 DIV. **M** Quantification of percent area change on 1 DIV relative to 0 DIV and normalized to control. Error bars represent SEM. Data was analyzed the non-parametric Mann–Whitney test. ns not significant, *p < 0.05, **p < 0.01 *n* = 4–6 mice per genotype from at least two independent litters. Scale bars represent 100 μm (**D**, **E**), 200 μm (**I**, **J**). DIV day in vitro, NPC neural precursor cells, NS neurospheres, 4OH-TMX 4-hydroxytamoxifen, P postnatal day, SVZ subventricular zone

For primary neurosphere migration analysis, 5–10 spheres were plated in each PDL- and laminin-coated well in a 24-well dish in triplicate. Adhered spheres were imaged as described above at 3 h post plating (0 DIV, prior to migration) and again after 24 h (1 DIV, after migration). Area of each sphere was outlined and quantified using ImageJ. Percent sphere area change from 0 to 1 DIV was calculated using the biological average and normalized to its littermate control. Each data point in Fig. 5L, M represents an average of a 12–48 individual primary neurospheres measured per each biological replicate from 3 independent litters. Representative Ankrd11^control^ and Ankrd11^nscKO^ 0 DIV and 1 DIV primary neurosphere area overlays are shown in Fig. 5K.

For scratch analysis, cells were plated on PDL- and laminin-coated well in a 24-well dish in triplicate and imaged as above. 0 DIV scratch boundary was overlayed onto 1 DIV and 2 DIV of the same well and region, cells within the scratch boundary were quantified using ImageJ. Number of migrated cells were quantified and normalized to a littermate control in each experiment.

**Fig. 6:**
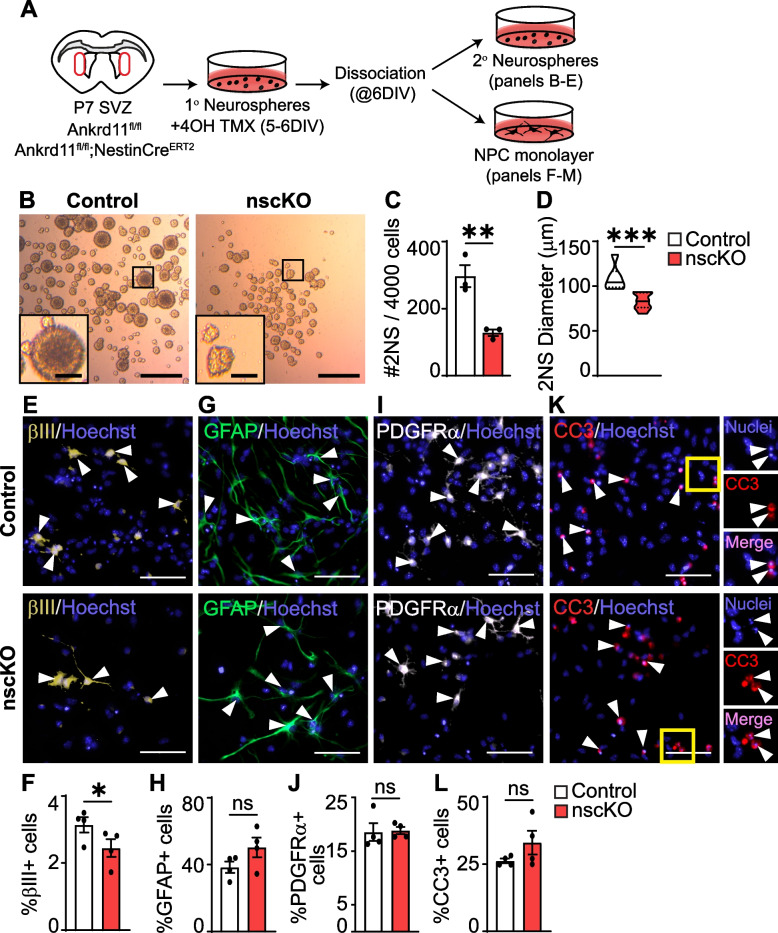
Loss of *Ankrd11* decreases SVZ NPC proliferation and causes aberrant neurogenesis in vitro. **A** Schematic: P7 SVZ primary neurospheres were cultured from Ankrd11^fl/fl^ and Ankrd11^fl/fl^; NestinCre^ERT2^ pups for 6 DIV. Neurospheres were treated with 4OH-TMX for 24 h prior to dissociation on 6 DIV resulting in Control and nscKO NPCs. Primary neurospheres were dissociated and further cultured as secondary neurospheres for 7 DIV or adherent NPC monolayer for 4 DIV. **B **Representative images of Ankrd11^control^ and Ankrd11^nscKO^ secondary neurospheres at 7 DIV. Inset is shown at the bottom left corner in higher magnification. **C**, **D** Quantification of **B** for secondary neurosphere number (**C**) and diameter (**D**). **E–L** Representative images and quantification of Ankrd11^control^ and Ankrd11^nscKO^ 4 DIV NPC monolayers immunostained for βIII (yellow) (**E**, **F**), GFAP (green) (**G**, **H**), PDGFRα (white) (**I**, **J**), CC3 (red) (**K**, **L**). Arrowheads indicate marker + cells. Yellow insets in **K** are shown at higher magnification on the right side. Cells were counterstained with Hoechst (blue). Data are presented as % marker + cells from healthy (non-condensed nuclei) except % CC3 + cells is presented as % from all nuclei (healthy and condensed). Examples of condensed nuclei positive for CC3 are shown in **K** and insets (yellow boxes) on right side. Error bars represent SEM. Data was analyzed using unpaired *t*-test. ns not significant, *p < 0.05, **p < 0.01, ***p < 0.001. *n* = 3–4 mice per genotype from at least two independent litters. Scale bars represent 500 μm (**B**), 100 μm (inset of **B**), 50 μm (**E**,** G**, **I**, **K**). DIV day in vitro, NPC neural precursor cells, NS neurospheres, 4OH-TMX 4-hydroxytamoxifen, P postnatal day, SVZ subventricular zone

All representative immunohistochemisty (IHC) and immunocytochemistry (ICC) images were enhanced post-acquisition linearly except representative images for DCX, PDGFRα, βIII, and GFAP stains (Figs. 2I, Q, S and 6E, G, I) were enhanced non-linearly to visualize cell processes.

#### Statistical analysis

All data passed normality test using the Kolmogorov–Smirnov or Shapiro–Wilk tests, except for the data from migration assays (Fig. 5), for which we performed a non-parametric Mann–Whitney test. For normal data, parametric tests were performed as following. For two-group comparisons, two-tailed unpaired Student’s or multiple *t*-tests were used to assess statistical significance between means. For three or more group comparisons, one- or two-way unpaired ANOVA was followed by Tukey’s multiple comparisons post hoc tests. In all cases, GraphPad Prism (version 8.0.2) was used for statistical tests. In the figures, an asterisk marks statistical significance indicated by *p-value of < 0.05, **p < 0.01, ***p < 0.001.

## Results

### Loss of Ankrd11 in NPCs results in aberrant postnatal OB development

Ankrd11 is expressed in both NPCs and neurons during embryonic brain development [38]. To determine the role of Ankrd11 in OB development, we induced knockout of *Ankrd11* in embyronic day (E) 14.5 NPCs and their subsequent progeny by breeding Ankrd11^fl/fl^ mice, where exon 7 of the *Ankrd11* gene is flanked by loxP sites, with TMX-inducible NestinCre^ERT2^ recombinase that is expressed specifically in Nestin + NPCs (Fig. 1A) [61–63]. Upon TMX administration, Cre causes recombination at the floxed exon 7 of *Ankrd11* in NPCs, creating a frameshift mutation that encompasses all known functional domains of the protein [58, 59]. The progeny from this TMX-injected cross that contain NestinCre^ERT2^ will be referred to as Ankrd11^nscKO^, and litter mates that do not contain a NestinCre^ERT2^ will be referred to as Ankrd11^Contol^ (Fig. 1A). To confirm loss of *Ankrd11* in NPCs, we dissected E15.5 Ankrd11^nscKO^ and Ankrd11^Contol^ neocortices 24 h post TMX injection. mRNA from cortical NPCs, which were enriched via neurosphere protocol [67], was subjected to qRT-PCR analysis. Figure 1K shows a ~ 96% decrease (*p* < 0.0001) in *Ankrd11* expression in Ankrd11^nscKO^ NPCs compared to the Ankrd11^control^ NPCs.

In mice, E14.5 is a timepoint that precedes the formation or migration of the majority of interneurons, glial cells, and glutamatergic cells in the developing OB [13]. Thus, for all in vivo experiments, Ankrd11^nscKO^ was induced at E14.5. To identify if the loss of *Ankrd11* in E14.5 NPCs influenced OB development, we first determined any gross changes in the OB at various endpoints using low-resolution photographs. At E18.5 (~ 1 day before birth) OBs in the Ankrd11^nscKO^ mice were visually indistinguishable from Ankrd11^control^ (Fig. S1B-D). Analysis of postnatal animals revealed that Ankrd11^Control^ OB continued to increase in size with age, as expected (Fig. 1B–J). While at P5, the gross OB size was comparable between Ankrd11^nscKO^ and Ankrd11^Control^ (Fig. 1B–C), starting from P10 and until P30 (latest timepoint analyzed), Ankrd11^nscKO^ OB was ~ 37–56% smaller compared to Ankrd11^Control^ OB (Fig. 1D–J). Notably, these changes were not due to non-specific effects of Cre-recombinase or TMX as OB area between uninjected Ankrd11^fl/fl^ and Ankrd11^fl/fl^;NestinCre^ERT2^ mice was not different and comparable to TMX-injected Ankrd11^Control^ (Fig. 1I–J). Importantly, OB area from P30 TMX-injected Ankrd11^nscKO^ animals was still decreased when compared to uninjected controls (Fig. 1J).

### Loss of Ankrd11 in NPCs does not affect OB development at E18.5 in vivo

To determine the effect of *Ankrd11* loss on OB development in more detail, we first analyzed the resultant E18.5 OB tissue following *Ankrd11* nscKO at E14.5. Analysis of the whole OB area (excluding ONL) showed no difference between E18.5 Ankrd11^nscKO^ and Ankrd11^control^ anatomically matched sagittal sections (Fig. S1A-D). To identify the center of the OB in an unbiased manner, we overlayed a crosshair pattern over each anatomically matched OB section. We then applied a circle that would encompass OB borders. Finally, we applied the same size column (rectangle), which originated from the OB center and emanated outwards in a rostral direction (towards circle edge), so all OB layers were captured (Fig. S1E). Resultant OB column boxes allowed us to analyze layer depth and cellular composition. Subsequent analysis of the OB neuronal layers, which were identified by Hoechst staining and as indicated in Fig. S1E-F, showed no changes in layer depth (Fig. S1G). Next, we analyzed the presence of NPCs and OB neurons. During the late embryonic stage, the majority of the M/T neurons and some interneurons have formed and migrated to their specific OB layers [13, 18]. Serial sagittal sections of E18.5 Ankrd11^nscKO^ and Ankrd11^control^ brains were immunostained for markers expressed in precursors and postmitotic neuronal cell types prevalent to OB development, including SOX2 (SRY-box transcription factor 2, a marker of NPCs [78]), DCX (doublecortin, marker of neuroblasts and immature neurons [79]) as well as NEUN (neuronal nuclear protein, a marker of excitatory and inhibitory neurons), CALB (calbindin, marker of periglomerular interneurons), and NG (neurogranin, marker of granule cell inhibitory interneurons) [15, 80, 81]. The identification of individual OB layers was determined by Hoechst nuclear staining and is indicated in Fig. S1F. We found no significant changes in the number of SOX2 + , DCX + , NEUN + , CALB + , and NG + cells between Ankrd11^control^ and Ankrd11^nscKO^ OB layers (Fig. S1H-Q). Finally, there were no changes between number of OPCs, detected using PDGFRα (platelet-derived growth factor receptor alpha) specific antibody [82], in any layer (Fig. S1R-S). Together, these data suggest that the knockout of *Ankrd11* in embryonic NPCs does not affect OB cell development at E18.5.

### Loss of Ankrd11 in NPCs causes aberrant OB development at P15 in vivo

Figure 1 demonstrated that OB in the Ankrd11^nscKO^ mice was significantly reduced after P10 and at least until P30. Therefore, we chose to perform an in-depth analysis of OB cell development at P15, a timepoint when postnatal interneuron genesis and glial cell proliferation is ongoing [2, 13]. Comparison of Ankrd11^control^ and Ankrd11^nscKO^ P15 OBs showed a ~ 38% reduction (*p* < 0.001) in the whole OB area (excluding ONL) (Fig. 2A–D). Next, we immunostained tissue sections for markers of neurons and glia. As in Fig. S1, we applied a circle and crosshairs method to determine OB center in an unbiased manner (Fig. 2E). Resultant OB column permitted analysis of layer depth and cellular composition. The identification of individual OB layers was determined by Hoechst nuclear staining and is indicated in Fig. 2F, G. Overall, we found a ~ 33% decrease (*p* < 0.0001) in the depth of the Ankrd11^nscKO^ GCL layer when compared to Ankrd11^Control^, resulting in a ~ 16% overall decrease of OB column (total layers) (*p* < 0.0001) in the Ankrd11^nscKO^ samples (Fig. 2H). We next analyzed cellular composition of each layer. Ankrd11^nscKO^ OB showed a ~ 42% (*p* < 0.001) reduction in total DCX + cells (Fig. 2I, J). This was due to a ~ 51% (*p* < 0.0001) decrease of DCX + cells in the GCL (Fig. 2I, J). This was accompanied by ~ 26% decrease (*p* < 0.0001) in total NEUN + cells due to a specific ~ 24% decrease (*p* < 0.0003) in NEUN + cells within the GCL (Fig. 2K, L). Notably, NEUN + cells in the Ankrd11^nscKO^ MCL and GCL exhibited a diffuse pattern of organization as compared to a well-defined pattern in Ankrd11^Control^ (Fig. 2K). This may indicate abnormal localization of neurons in the Ankrd11^nscKO^ mice, which supports and extends prior reports that demonstrated aberrant cortical neuronal localization in the Ankrd11-deficient mice [38, 83]. Finally, Ankrd11^Control^ and Ankrd11^nscKO^ samples showed comparable CALB + , NG + , GFAP + (glial fibrillary acidic protein), an astrocyte marker [84], and PDGFRα + cells in all OB layers (Fig. 2M–T). In summary, our results demonstrate a smaller OB size in P15 Ankrd11^nscKO^ mice accompanied by a specific decrease in DCX + and NEUN + cells in the GCL and reduced GCL size. Therefore, the OB size in the Ankrd11^nscKO^ is most probably reduced due to aberrant formation and/or maintenance of the GCL.

### Loss of Ankrd11 in NPCs results in aberrant postnatal RMS development in vivo

Postnatal OB development and maintenance are dependent on the rostral migratory stream (RMS) [13, 18, 76]. DCX + neuroblasts originating in the SVZ travel along the RMS during postnatal development and in adulthood via a GFAP + astrocyte network into the OB, where they differentiate and integrate into OB outer neuronal layers, thus contributing to OB growth [2, 16, 22]. To ask whether the RMS in the Ankrd11^nscKO^ brains is perturbed, we immunostained serial E18.5 and P15 sagittal sections of Ankrd11^control^ and Ankrd11^nscKO^ brains for DCX, GFAP, and SOX2 (Figs. S2 and 3).

In the E18.5 sections, DCX stained all neurons in the brain as they represent immature neurons (data not shown). To identify RMS, we instead used SOX2 staining, which at this timepoint is restricted to select areas including a thin layer of cortical SVZ and RMS (Fig. S2B). SOX2 + cells formed a continuous stream of cells that connect to the OB in both Ankrd11^nscKO^ and Ankrd11^control^ E18.5 brains (Fig. S2B). Integrated density analysis showed no significant changes in the SOX2, DCX, or GFAP fluorescence levels in the RMS (Fig. S2C–E). There were also no gross apparent changes in RMS integrity at this timepoint (Fig. S2B).

In contrast to E18.5, RMS in the P15 Ankrd11^nscKO^ brain sections appeared to be interrupted between the SVZ and OB (indicated by red arrows in Fig. 3A-D). This was apparent in images with DCX immunostaining and nuclear (Hoechst) staining (Fig. 3C, D). To quantify this and explore the potential consequences on the downstream migration and population within the OB, we measured the nuclei density from three locations along the RMS (Fig. 3E). Region 1 encompassed the caudodorsal (CD) RMS (indicated by blue arrowheads in Fig. 3C, D) and region 2 measured the ventral intermediate (VI) zones (indicated by red arrows in Fig. 3C, D), which were identified and demarcated according to [75, 76]. Region 3 sampled the area where the RMS connects to the OB (indicated by yellow star in Fig. 3C, D). Integrated density analysis of the nuclear stain Hoechst revealed a ~ 45% and ~ 25% significant decreases (*p* < 0.05) in cell density in Ankrd11^nscKO^ regions 2 and 3, respectively (Fig. 3E). While the density of nuclei was not changed in a defined region of interest inside region 1 of RMS, the size of the whole region 1 of the Ankrd11^nscKO^ RMS area was ~ 70% (*p* = 0.008) increased when compared to Ankrd11^control^, further highlighting the aberrant RMS (Figs. 3F, S3A,C). Together, this indicates aberrant RMS development in the Ankrd11^nscKO^ brain.

Next, we asked if Ankrd11^nscKO^ RMS displayed aberrant cell proliferation, differentiation, and/or apoptosis. First, mice were injected with BrdU 24 h prior to perfusion to label actively proliferating cells (Fig. 4A). RMS was identified with neuroblast-specific anti-PSA-NCAM (polysialylated neuronal cell adhesion molecule), which completely overlaps with DCX (data not shown) [85]. Analysis of region 1 (caudodorsal RMS adjacent to LV) (Figs. 4B, S3A) revealed that the number and density of BrdU + cells was ~ 76% (*p* < 0.0001) decreased in Ankrd11^nscKO^ when compared to Ankrd11^Control^ (Fig. 4C–E). This was not due to a decrease in progenitors as the number and density of SOX2 + cells and integrated density of PSA-NCAM was similar between Ankrd11^nscKO^ and Ankrd11^Control^ (Figs. 4F, G, S3B). However, the number, density, and proportion of proliferating BrdU + SOX2 + cells were decreased by ~ 50% (*p* < 0.01) in Ankrd11^nscKO^ when compared to Ankrd11^Control^ (Fig. 4H–J). Next, we asked whether Ankrd11^nscKO^ RMS displayed aberrant cell apoptosis. Analysis of region 1 using anti-CC3 (cleaved caspase 3), an apoptosis marker [63], revealed no differences in the number or density of CC3 + cells between Ankrd11^nscKO^ and Ankrd11^Control^ (Fig. 4K-M). Thus, Ankrd11^nscKO^ progenitors in the RMS display reduced proliferation, which supports and extends previous reports showing reduced proliferation of Ankrd11-deficient cortical progenitors [38].

The RMS is populated by migrating progenitors that terminally differentiate into postmitotic interneurons once they reach the OB [86]. However, overexpression or knockout of NeuroD1 or NCAM, respectively, have resulted in RMS with prematurely differentiated neurons [87, 88]. We thus asked whether Ankrd11^nscKO^ RMS contained any prematurely differentiated neurons. We analyzed RMS region 1, which was identified by PSA-NCAM staining (Fig. S3C). We observed a > 2.8-fold increase in the number and density (*p* = 0.001) of NEUN + cells in the Ankrd11^nscKO^ RMS when compared to Ankrd11^Control^ (Fig. 4N–P). These results suggest a precocious differentiation of RMS progenitors into mature neurons before they can reach the OB and integrate into the GCL. These observations support and extend previous reports that demonstrate precocious neuronal differentiation in progenitor-rich and usually neuron-devoid cortical SVZ in mice with *Ankrd11* knockdown or mutations [38].

Together, these results indicate an apparently normal RMS in embryonic Ankrd11^nscKO^ mice and a discontinuous RMS in postnatal Ankrd11^nscKO^ mice, which may suggest aberrant progenitor migration. Moreover, our results indicate a decrease in RMS progenitor proliferation and aberrant differentiation. To corroborate and tease apart the roles of Ankrd11 in migration, proliferation, and differentiation, we pursued in vitro cultures.

### Loss of Ankrd11 results in decreased migration of SVZ NPCs in vitro

To confirm the effect on migration of SVZ-derived Ankrd11^nscKO^ NPCs, we cultured P7 SVZ NPCs from Ankrd11^fl/fl^ and Ankrd11^fl/fl^;NestinCre^ERT2^ pups that were not injected with TMX, using a neurosphere protocol for 6 DIV (days in vitro) [62, 67]. We have previously shown that these neurospheres are highly enriched in Nestin + NPCs [67]. Recombination in primary neurospheres was induced by adding 4OH-TMX (active component of TMX) to the media for 24 h prior to downstream migration assays (Fig. 5A). RT-qPCR analysis of 4-OHT treated neurospheres revealed ~ 80% *Ankrd11* mRNA reduction (*p* = 0.012) in Ankrd11^nscKO^ cultures when compared to Ankrd11^Control^ (Fig. 5B).

First, we dissociated 4-OHT treated primary neurospheres from Ankrd11^nscKO^ and Ankrd11^Control^ cultures and plated them in a high-density monolayer. We then performed a scratch assay and assessed cell migration immediately after scratch formation and 1 and 2 DIV thereafter (Fig. 5C). Ankrd11^control^ cells gradually filled in the scratch area over 2 DIV (Fig. 5D). In contrast, Ankrd11^nscKO^ cells showed ~ 51–64% decrease in the number of migrated cells on 1 and 2 DIV, respectively (*p* < 0.01), when compared to Ankrd11^Control^ (Fig. 5E-G).

We have corroborated these results with a neurosphere migration assay [69, 89]. Here, individual 4OH-TMX treated primary neurospheres were plated without dissociation onto PDL- and laminin-coated wells; the migration of cells out of the adhered spheres was assessed at the time of plating and after 1 DIV (Fig. 5H). Migration was determined by analyzing the change in total neurosphere area from 0 to 1 DIV (Fig. 5I–K). At the time of plating (0 DIV), neurosphere area was similar between Ankrd11^control^ and Ankrd11^nscKO^ (Fig. 5I–L). On 1 DIV, Ankrd11^control^ neurospheres were visually more uniform creating a flattened surface of migrated NPCs (Fig. 5I), while Ankrd11^nscKO^ neurospheres remained as a dense sphere with decreased individual cells migrating out (Fig. 5J). Quantification showed a ~ 34% decrease in Ankrd11^nscKO^ neurosphere area change relative to Ankrd11^control^ (*p* = 0.0095) on 1 DIV (Fig. 5K, M).

Together, these results show that loss of Ankrd11 leads to decreased NPC migration in vitro.

### Loss of Ankrd11 leads to reduced NPC proliferation and neurogenesis in vitro

To corroborate the effect of Ankrd11 deficiency on NPC proliferation, we first generated primary neurospheres from P7 Ankrd11^fl/fl^ and Ankrd11^fl/fl^;NestinCre^ERT2^ SVZ NPCs and treated them with 4OH-TMX to induce recombination (Fig. 6A and as described above). Twenty-four hours after 4OH-TMX treatment, these spheres were dissociated and plated at clonal density for 7 DIV to form secondary neurospheres, which were used as a readout of NPC proliferation [77]. Analysis of secondary neurospheres revealed a ~57% decrease (*p* = 0.0078) in the number and ~24% decrease in the diameter (*p* = 0.0002) of Ankrd11^nscKO^ spheres compared to Ankrd11^control^ (Fig. 6B–D). These results show that Ankrd11^nscKO^ NPCs have reduced proliferation in vitro (Fig. 6B–D) and corroborate our in vivo results (Fig. 4C–J).

To determine whether Ankrd11 regulates NPC differentiation, we dissociated 4OH-TMX treated primary neurospheres and plated them as adherent monolayers at a density that we have previously showed was optimal for differentiation into neurons and glia by 4–5 DIV [67] (Fig. 6A). Immunostaining on 4 DIV revealed a ~ 22% decrease in the formation of βIII + neurons (*p* = 0.0095) in the Ankrd11^nscKO^ cultures when compared to Ankrd11^Control^ (Fig. 6E, F) without significant effect on GFAP + astrocytes, PDGFRα + oligodendrocyte precursor cells, or apoptotic CC3 + cells (Fig. 6G–L). Therefore, loss of Ankrd11 results in decreased neurogenesis in vitro.

### OB anomalies are detected in KBG syndrome patients

**Fig. 7:**
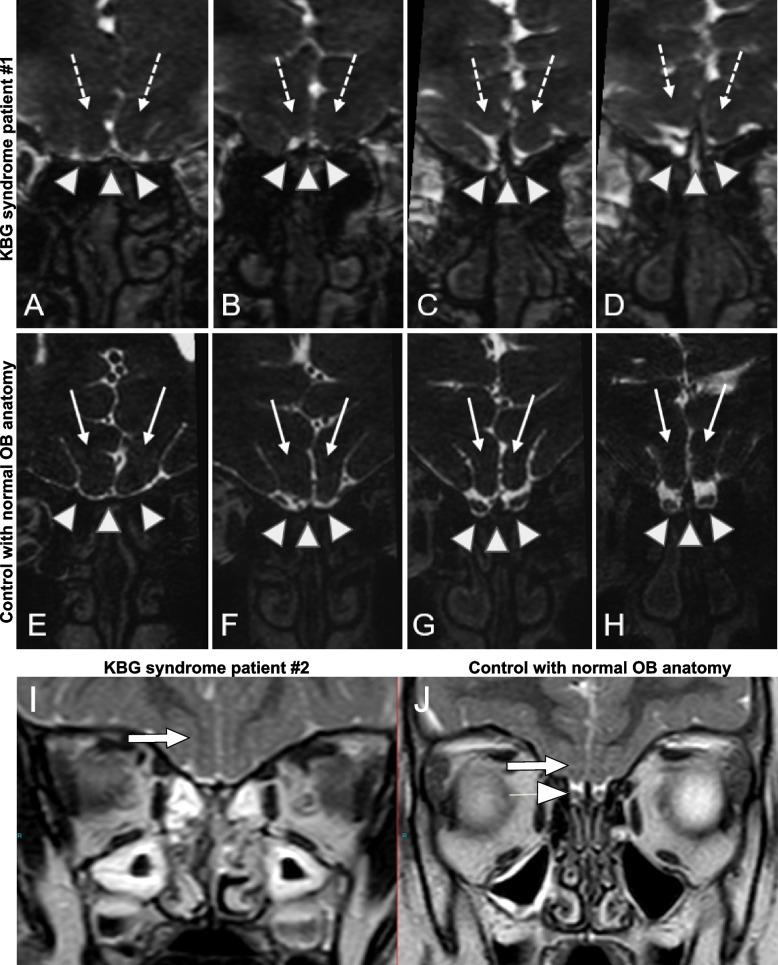
OB hypoplasia in KBG syndrome patients. Coronal multiplanar reformatting three-dimensional high resolution heavy T2-weighted images of the naso-ethmoidal region. **A–D** Patient #1; MRI at 14 years of age. **E–H** Normal control anatomy. The olfactory bulb is not clearly visible in KBG syndrome patient as compared to the normal subject (arrowheads). In addition, the olfactory grooves are severely hypoplastic and the straight gyri (dotted arrows in **A–D**) are dysmorphic with less prominent and organized adjacent sulci with respect to the normal anatomy (arrow in **E–H**). **I** Patient #2; MRI at 3 years of age. **J** Normal control anatomy. No olfactory bulbs are seen in the KBG syndrome patient and consequently there are no olfactory grooves. The olfactory sulci are visible (arrow). Olfactory bulbs (arrowhead) are visible within well-formed olfactory grooves of the control. The olfactory sulci (arrow) can also be seen

An MRI scan of patient #1 (male; de novo heterozygous variant c. 3221_3222delAA, p. Lys1074ArgfsTer27 in the *ANKRD11* gene) showed normal brain structures; particularly, the sella turcica and hypophysis were morphologically normal, but the olfactory bulbs were not clearly depicted compared to normal control images. The olfactory grooves looked severely hypoplastic and the straight gyri had a dysmorphic aspect with less prominent and organized adjacent sulci as in normal anatomy (Fig. 7A–H). These observations were interpreted as dysgenesis or hypoplasia of the olfactory bulbs and nerves, possibly associated with the clinically confirmed anosmia. Patient #2 (female; c.6152dupC de novo in the *ANKRD11* gene) came to genetic counseling for psychomotor retardation and olfactory bulb agenesia. Her olfactory bulbs were not seen on cerebral MRI confirming OB hypoplasia, dysplasia, or agenesis (Fig. 7I-J). Other relevant clinical observations included failure to thrive for patient #2 and relatively poor response to growth hormone therapy in patient #1. While patient #2 has psychomotor retardation and severe speech delay, patient #1 is cognitively average and was able to complete school education. Finally, both patients present with mood disorders, including depressive behavior, anxiety, frustration intolerance, and/or anger.

We have also performed a Facebook KBG foundation poll, which was answered by 103 families; 73 reported normal sense of smell with 21 stating that it was not possible to know if the sense of smell was normal. Notably, 9 (8.7%) reported alterations to sense of smell, with 5 (4%) reporting absent sense of smell, and 4 (3.8%) reporting a reduced sense of smell.

Together, these results identify anosmia, hyposmia, olfactory nerve and/or OB dysgenesis or hypoplasia as novel clinical symptoms of KBG syndrome.

## Discussion

In this study, we show that conditional ablation of *Ankrd11* in murine embryonic NPCs results in abnormal postnatal OB development, including abrogated RMS, reduced GCL, and overall smaller OB size. We further show Ankrd11-deficient NPCs display reduced proliferation, migration, and neurogenesis. Finally, we also show two KBG syndrome patients with clinical anosmia and/or OB hypoplasia. Thus, ANKRD11 is required for proper olfactory bulb development in mice and humans.

Several transcription regulators and signaling receptors are critical for tangential neuroblast migration in the RMS and subsequent OB development. For example, NFIX (Nuclear Factor IX) constitutive knockout mice display enlarged SVZ, reduced RMS, and postnatal OB hypoplasia [90]. Conditional knockout of Tbr2 (T-box transcription factor 2) in E11 NPCs results in stunted embryonic and postnatal OB development at least in part due to enlarged cellular density in and size of the RMS as early as E16.5 [91]. Furthermore, conditional knockout of Mll1 (mixed lineage leukemia protein-1) in E13.5 NPCs leads to postnatal OB hypoplasia in vivo, reduced neuroblast density in the RMS ex vivo as well as diminished neuroblast chain migration from SVZ neurospheres in vitro [92]. Finally, constitutive knockout of Prokr2 (prokineticin receptor 2), which binds PROK2, a chemoattractant for neuroblasts in the RMS [93], results in fewer neuroblasts in the OB in vivo and an impaired migration of SVZ NPCs towards Prok2 in vitro [94]. In agreement, Sp8/Sp9 knockout mice show aberrant RMS migration and OB development at least in part via downregulation of the Prokr2 expression [95]. Our results showing that ablation of *Ankrd11* in NPCs leads to reduced NPC migration in vitro and aberrant RMS development in vivo support and extend these reports.

Notably, we show a distinct OB phenotype compared to studies cited above. Constitutive or NPC-specific knockouts of NFIX, Tbr2, Mll1, or Prokr2 lead to gross changes in all OB layers, including GCL [90–95]. However, we show that *Ankrd11* conditional knockout in embryonic NPCs leads to reduced neuroblasts and postmitotic neurons only in the GCL. The inhibitory granule neurons in the GCL are one of the most abundant neuron types in the OB and play a critical role in the discrimination of odors through their synaptic interactions with the excitatory M/T neurons located in the MCL [96, 97]. Notably, our results demonstrate abnormal neuronal localization in the GCL and MCL of the Ankrd11^nscKO^ OB. We suspect this could be due, at least in part, to the aberrant neuronal migration. This is supported by our in vitro results that show decreased progenitor migration*.* It is tempting to speculate that these changes may have a direct impact on olfaction. While we did not perform behavioral olfaction experiments, our clinical data align well with murine results and demonstrate absence or hypoplasia of OB and OB-associated structures co-present with anosmia/hyposmia in two KBG syndrome patients.

Contribution of migrating postnatal neuroblasts in the RMS to postnatal and adult OB development and function is supported by prior literature [2]. For example, sensory deprivation via naris occlusion (nasal cavity blockage) leads to reduced neuroblast migration in the RMS and stunted OB growth, while removal of the naris occlusion restores neuroblast migration and OB development [98]. Notably, the restoration of OB growth in olfaction deprived mice requires neuroblast formation from SVZ NPCs [98]. Mouse genetic models demonstrate that proper RMS and OB development is also regulated by several neurogenesis-related genes. For example, DCX deficiency leads to abnormal SVZ NPC migration, precocious differentiation in the RMS, and a smaller OB due to loss of Calretinin + granule cells [88, 99]. Overexpression of NeuroD1, a neurogenic transcription factor, induces premature differentiation of neurons in RMS [87]. Our data suggest that the reduction in OB size in the Ankrd11^nscKO^ mice is due to reduced NPC proliferation, migration, and aberrant neurogenesis. Notably, while the Ankrd11^nscKO^ OB GCL contained reduced number of neurons, the RMS contained more mature neurons when compared to control. It is likely that the Ankrd11-deficient progenitors that reached the OB have reduced neurogenesis, while progenitors that are present in the abrogated RMS differentiate locally before reaching the OB, similarly to DCX-deficient mice.

We and others have previously shown aberrant neuronal positioning in the cortex of mice that carry a splice mutation in the *Ankrd11* gene (Yoda mice) or in mice with *Ankrd11* knockdown in murine embryonic cortical NPCs, which could suggest aberrant neuronal migration [38, 83]. Our results extend and support these conclusions by showing neuronal mislocalization (heterotopia) in the Ankrd11^nscKO^ RMS. Together, this suggests that some Ankrd11 mechanisms may be shared between OB and cortical development. Furthermore, our report is the first to show that Ankrd11 deficiency in NPCs causes severe migration defects via the abrogated postnatal RMS in vivo and reduced migration in vitro.

From a clinical perspective, this is the first case of a clear link between ANKRD11, KBG syndrome, and OB deficits. Previously, one patient with 16q24.3 microdeletion, which encompasses *ANKRD11*, was reported to have anosmia [100]. Since clinical phenotypes between patients with *ANKRD11* variants vs 16q24.3 microdeletions may vary in nature, numbers, penetrance, and severity [100], it was not clear whether the anosmia in the 16q24.3 microdeletion patient was due to the deletion of *ANKRD11* and/or any other genes. Our report showing OB deficiencies in two patients with *ANKRD11* variants as well as clear OB developmental reduction in size in mice lacking *Ankrd11* in embryonic NPCs and their derivatives establishes a strong causative link between *ANKRD11* perturbations and OB deficiencies. Hypo/anosmia is not a phenotype that would routinely be enquired about or reported. Indeed, if one has congenitally reduced sense of smell one may never know that it is reduced. To this end, the Facebook survey on sense of smell was responded to by 103 families with KBG syndrome patients, which represents approximately a quarter of known families registered with the KBG foundation. Of these 8.7% reported reduced or absent sense of smell. Notably, ~ 29% of respondents stated that it was not possible to know if the sense of smell was normal. Our report demonstrates OB defects in two KBG syndrome patients, and this along with the results of the poll suggest this may be a relatively rare feature in KBG syndrome or an under recognized/reported one.

OB malformations, including uni- and bilateral OB hypoplasia or aplasia, are reported in ~ 30% of patients with Mendelian Disorders of the Epigenetic Machinery [101]. Notably, chromatin or epigenetic regulation represents the largest group of NDD risk genes [102–104]. Not surprisingly, OB malfunctions, which include anosmia (loss of smell), hyposmia (reduced scent detection), and hyperosmia (enhanced scent detection) or OB dysplasia have been detected in patients with ASD [105–108], including patients with variants in epigenetic regulators like CBP (cyclic AMP response element binding protein binding protein; Rubinstein–Taybi syndrome risk gene) and FMR1 (fragile X messenger ribonucleoprotein 1; fragile X Syndrome risk gene) [109–111]. Our results showing absent or altered OB with/without anosmia in KBG syndrome patients and reduced postnatal OB size and altered RMS development in the Ankrd11^nscKO^ mice support and extend these reports suggesting that epigenetic risk genes of developmental disorders may be key regulators of OB development and function.

The sense of smell is strongly connected to the emotional processing via the connection of the olfactory system with reward pathways and the hippocampus [8, 112]. Reductions in the ability to smell or OB ablation can drastically change the food intake [113, 114]. For example, OB ablation results in altered feeding patterns in rats and sheep [115, 116]. Human patients with traumatic brain injury that show changes in olfaction and the OB have difficulties in feeding [117]. With regard to behavior, reduction or loss of olfaction has close ties to behavioral changes in mood-altering disorders, and OB changes themselves can be used as a marker for depression [9, 10, 118]. Furthermore, OB bulbectomy or OB neuronal ablation in rodents induces depressive-like behaviors [6–8]. Notably, some patients with NDDs, including KBG syndrome, experience feeding difficulties and failure to thrive as well as frustration and depressive behavior [45, 51, 52, 119–121]. While the link between OB defects or malfunction and NDD-related behaviors is not currently known, our work and previous reports suggest a more systematic recording of sense of smell in newly diagnosed patients along with continued efforts to record the detailed feeding and neurobehavioral phenotypes may be warranted. While it is unlikely that hyposmia or anosmia would be amenable to clinical treatment, it may be important for appropriate genetic counseling.

## Conclusions

Taken together, our results demonstrate a novel and critical role for ANKRD11 in OB development and suggest OB size or olfaction evaluations should be considered upon KBG syndrome diagnosis for appropriate genetic counseling and to improve clinical care.

### Supplementary Information

**Supplementary Material 1.**

## Data Availability

All data generated or analyzed during this study are included in this published article and its supplementary information files.

## Abbreviations

4OH-TMX: 4-Hydroxytamoxifen
ADHD: Attention-deficit/hyperactive disorder
Ankrd11: Ankyrin repeat domain 11
ASD: Autism spectrum disorder
βIII: Beta-III tubulin
BrdU: Bromodeoxyuridine
BSA: Bovine serum albumin
CALB: Calbindin
CBP: CREB binding protein
CC3: Cleaved caspase 3
CD: Caudodorsal
CNS: Central nervous system
DCX: Doublecortin
DIV: Day in vitro
E: Embryonic day
ECM: Extracellular matrix
EGF: Epidermal growth factor
EPL: External plexiform layer
FGF: Fibroblast growth factor
FMR1: Fragile X messenger ribonucleoprotein 1
GCL: Granule cell layer
GFAP: Glial fibrillary acidic protein
GL: Glomerular layer
HAT: Histone acetyltransferase
HBSS: Hanks’ Balanced Salt Solution
HDACs: Histone deacetylase
HEP: Heparin
IP: Intraperitoneal
LV: Lateral ventricle
M/T: Mitral tufted cells
MCL: Mitral cell layer
MeCP2: Methyl CpG binding protein 2
MRI: Magnetic resonance imaging
NG: Neurogranin
NDD: Neurodevelopmental disorders
NPC: Neural precursor cells
NEUN: Neuronal nuclei
OB: Olfactory bulb
ONL: Olfactory nerve layer
OPC: Oligodendrocyte precursor cells
OSN: Olfactory sensory nerve
P: Postnatal day
PSA-NCAM: Polysialylated-neural cell adhesion molecule
PDGFRα: Platelet-derived growth factor receptor alpha
PDL: Poly-d-lysine
Prok2: Prokineticin 2
Prokr2: Prokineticin receptor 2
RMS: Rostral migratory stream
SOX2: Sex-determining region Y (SRY)-related high-mobility-group (HMG)-box 2
SVZ: Subventricular zone
TMX: Tamoxifen
VI: Ventral intermediate
