# Correction: Olfactory bulb anomalies in KBG syndrome mouse model and patients

**DOI:** 10.1186/s12916-024-03519-4

**Published:** 2024-07-11

**Authors:** Kara Goodkey, Anita Wischmeijer, Laurence Perrin, Adrianne E. S. Watson, Leenah Qureshi, Duccio Maria Cordelli, Francesco Toni, Maria Gnazzo, Francesco Benedicenti, Monique Elmaleh‑Berges, Karen J. Low, Anastassia Voronova

**Affiliations:** 1 Department of Medical Genetics, Faculty of Medicine & Dentistry, University of Alberta, Edmonton, AB T6G 2H7 Canada; 2 Women and Children’s Health Research Institute, University of Alberta, 5‑083 Edmonton Clinic Health Academy, Edmonton, AB T6G 1C9 Canada; 3 Clinical Genetics Service and Coordination Center for Rare Diseases, Department of Pediatrics, Regional Hospital of Bolzano, Bolzano, Italy; 4 Clinical Genetics Unit, Hopital Robert-Debre, Paris, France; 5 IRCCS Istituto Delle Scienze Neurologiche Di Bologna, UOC Neuropsichiatria Dell’eta Pediatrica, Bologna, Italy; 6 Programma Di Neuroradiologia Con Tecniche Ad Elevata Complessita (PNTEC), IRCCS Istituto Delle Scienze Neurologiche Di Bologna, Bologna, Italy; 7 Laboratory of Medical Genetics, Translational Cytogenomics Research Unit, Bambino Gesu Children’s Hospital, IRCCS, 00165 Rome, Italy; 8 Service d’Imagerie Pediatrique, Hopital Universitaire Robert Debre, Paris, France; 9 Department of Academic Child Health, Bristol Medical School, Population Health Sciences, University of Bristol, Bristol, UK; 10 Clinical Genetics Service, St. Michaels Hospital, Bristol, UK; 11 Department of Cell Biology, Faculty of Medicine & Dentistry, University of Alberta, Edmonton, AB T6G 2H7 Canada; 12 Faculty of Medicine & Dentistry, Neuroscience and Mental Health Institute, Edmonton, AB T6G 2E1 Canada

**Correction: BMC Medicine 22, 158 (2024)**

**https://doi.org/10.1186/s12916-024-03363-6**

The original article contains two minor typos that the authors wish to address:

1) In the sentence, "Notably, 9 (8.7%) reported alterations to sense of smell, with 5 (4%) reporting absent sense of smell, and 4 (3.8%) reporting a reduced sense of smell.", ‘4%' should instead read as ‘4.9%', and ‘3.8%' should instead read as ‘3.9%'.

2) In the sentence, “Notably, ~ 29% of respondents stated that it was not possible to know if the sense of smell was normal.”, ‘29%’ should instead read as ‘20%’.

## References

[1] Goodkey K (2024). Olfactory bulb anomalies in KBG syndrome mouse model and patients. BMC Med.

