# Supplementary material for: Olfactory bulb anomalies in KBG syndrome mouse model and patients
Source: BMC Med. 2024 Apr 15;22:158. doi: 10.1186/s12916-024-03363-6 (PMC11017579; doi:10.1186/s12916-024-03363-6)
Supplement: Supplementary file 1 — Supplementary Material 1. [file 12916_2024_3363_MOESM1_ESM.pdf]

BMC Medicine

Supplemental Information

Olfactory bulb anomalies in KBG syndrome mouse model and patients

Kara Goodkey, Anita Wischmeijer, Laurence Perrin, Adrianne E. S. Watson, Leenah Qureshi, Duccio Maria Cordelli, Francesco Toni, Maria Gnazzo, Francesco Benedicenti, Monique Elmaleh-Bergès, Karen J. Low, and Anastassia Voronova

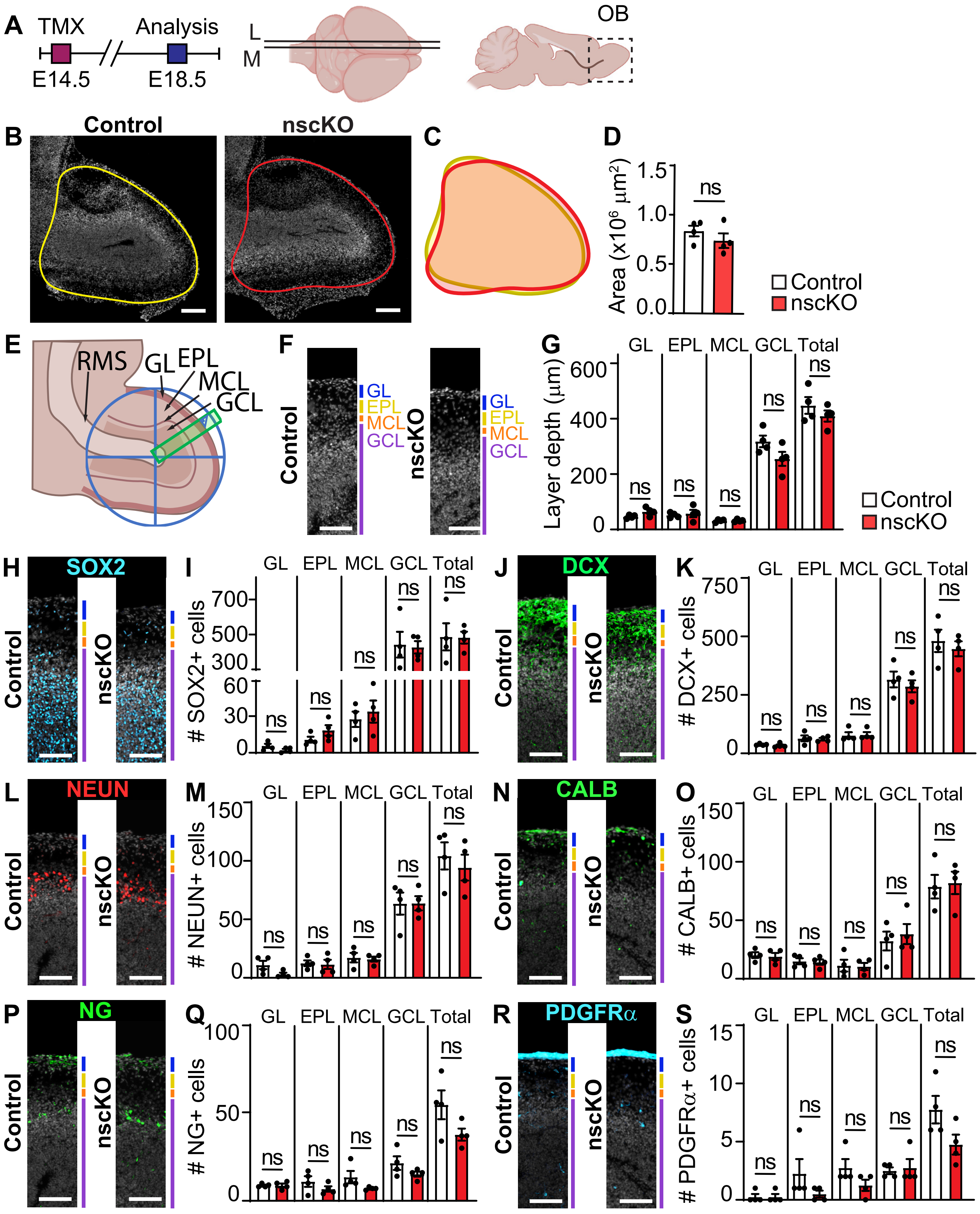

Fig. S1 (See legend on next page.)

Figure S1. Related to Figure 2. Loss of *Ankrd11* in E14.5 NPCs does not lead to changes in E18.5 OB development.

(A) Ankrd11$^{fl/fl}$;RosaYFP$^{STOP/STOP}$ dams mated with Ankrd11$^{fl/fl}$;RosaYFP$^{STOP/STOP}$;NestinCre^{ERT2} males were injected with TMX at E14.5. Resulting embryos (Ankrd11^{control} and Ankrd11^{nscKO}) were collected at E18.5. Images were collected from anatomically matched medial (M) and lateral (L) sagittal sections encompassing OB. **(B)** Representative images of Ankrd11^{control} and Ankrd11^{nscKO} sagittal sections stained for Hoechst (white). Solid outline shows OB area (excluding ONL) of Ankrd11^{control} (yellow) and Ankrd11^{nscKO} (red). **(C)** Representative overlap of Ankrd11^{control} and Ankrd11^{nscKO} OB areas from (**B**). **(D)** Quantification of (**B**). **(E)** Representative E18.5 OB schematic that displays identification of OB centre by circle and crosshairs method followed by demarcation of the OB column and layers. Please see "Materials and Methods" for more details. RMS=rostral migratory stream; GL = glomerular layer; MCL = mitral cell layer; EPL = external plexiform layer; GCL = granule cell layer. **(F)** Representative images of Ankrd11^{control} and Ankrd11^{nscKO} OB column crop is on the right side with demarcated layers. **(G)** Quantification of OB layer depth shown in **(F)**. **(H-S)** Representative images and quantification of marker+ cells in Ankrd11^{control} and Ankrd11^{nscKO} OB column layers immunostained for SOX2 (blue) (**H-I),** DCX (green) (**J-K**), NEUN (red) (**L-M**), CALB (green) (**N-O**), NG (green) (**P-Q**), PDGFRα (blue) (**R-S**). Layer demarcation indicated to the right. Hoechst in counterstained in white. The Hoechst image panels in **H** and **J** came from one multiplex image immunostained for SOX2 and DCX. The Hoechst image panels in **L** and **P** were obtained from a separate multiplex image immunostained for NEUN and NG. The Hoechst image panels in **N** and **R** came from one multiplex image immunostained for CALB and PDGFRα. Error bars represent SEM. Data were analyzed 2-way ANOVA followed by Tukey's multiple comparisons post-hoc test, except data in D were analyzed using unpaired t-test. ns=not significant. n = 4 mice per genotype from at least 2 independent litters. Scale bars represent 200 µm (B), 100 µm (F,H,J,L,N,P,R) E = embryonic day; L= lateral; LV – lateral ventricle; M = medial; OB = olfactory bulb; TMX = Tamoxifen.

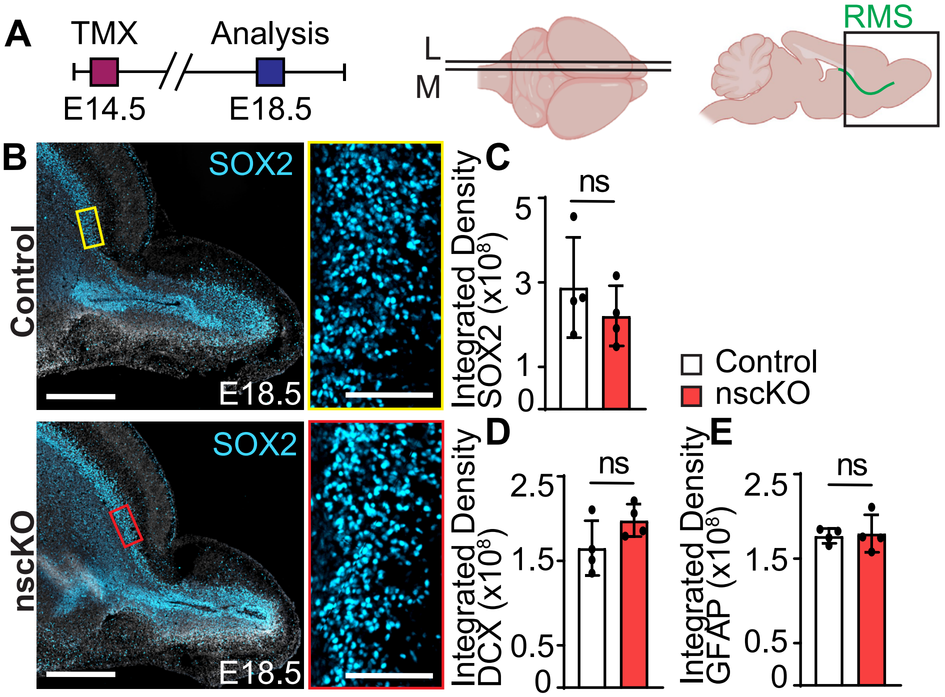

Figure S2. Related to Figure 3. Loss of *Ankrd11* in E14.5 NPCs does not lead to changes in E18.5 RMS.

(A) Ankrd11$^{fl/fl}$;RosaYFP$^{STOP/STOP}$ dams mated with Ankrd11$^{fl/fl}$;RosaYFP$^{STOP/STOP}$;NestinCre^{ERT2} males were injected with TMX at E14.5. Resulting mice (Ankrd11^{control} and Ankrd11^{nscKO}) were collected at E18.5. Images were collected from anatomically matched medial (M) and lateral (L) sagittal sections encompassing OB. RMS indicated in green. **(B)** Representative images of Ankrd11^{control} and Ankrd11^{nscKO} E18.5 OB and RMS immunostained for SOX2 (blue). Nuclei were counterstained with Hoechst (white). Boxes in yellow (Ankrd11^{Control}) and red (Ankrd11^{nscKO}) are shown as high magnification images in the right column and quantified in (**C-E**). **(C)** Quantification of (**B**), where integrated density of SOX2 within RMS (yellow or red box in [**B**]) is quantified as outlined in Materials and Methods section. **(D-E)** Integrated density analysis of DCX (**D**) and GFAP (**E**) signal in Ankrd11^{control} and Ankrd11^{nscKO} E18.5 RMS. Error bars represent SEM. Data was analyzed using unpaired t-test, ns=not significant. n=4 mice per genotype from at least two independent litters. Scale bars represent 500 μm (B), 100 μm (Inset of B). E = embryonic day; L= lateral; M = medial; OB = olfactory bulb; RMS = rostral migratory stream.

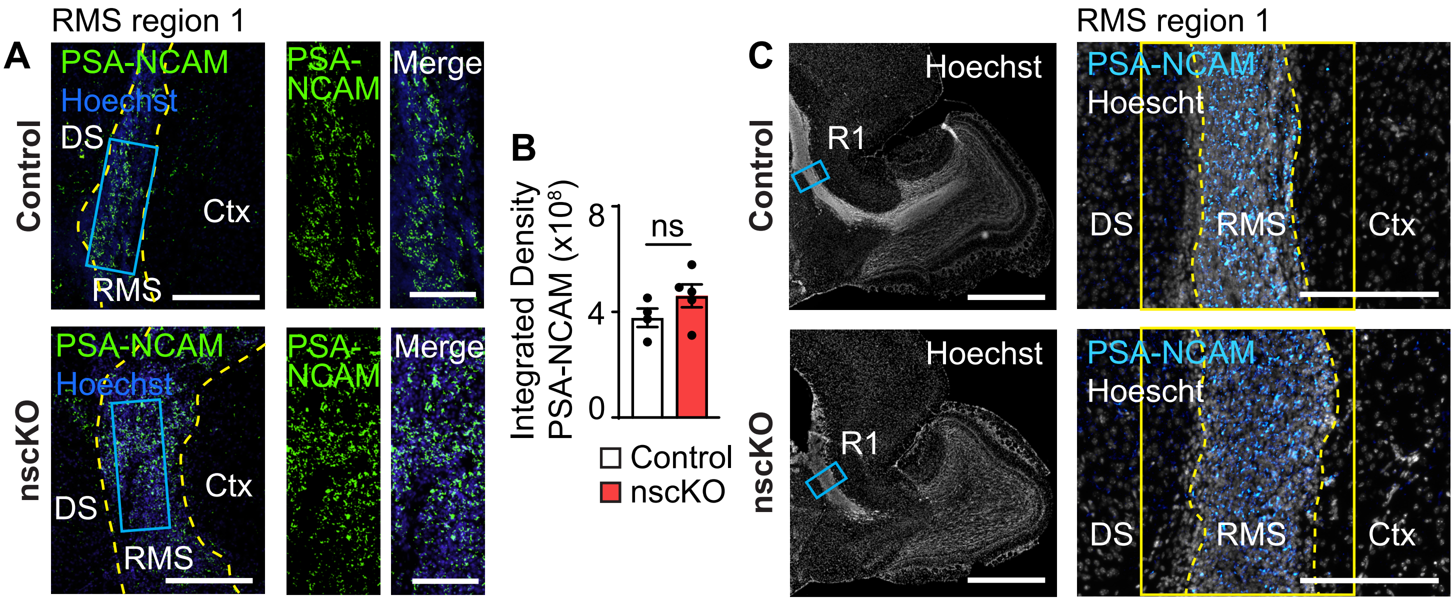

Figure S3. Related to Figure 4. Loss of *Ankrd11* in E14.5 NPCs does not affect density of neuroblasts in the P15 caudodorsal RMS.

(A) Representative images of $Ankrd11^{control}$ and $Ankrd11^{nscKO}$ of P15 RMS region 1 (caudodorsal [CD]) immunostained for PSA-NCAM (green) and Hoechst (dark blue). Yellow dashed line indicates RMS defined by PSA-NCAM boundaries and the same size blue box indicates area of region of interest used in BrdU analysis in Fig. 4C-J and is shown in higher magnification on the right side. **(B)** Quantification of integrated density of PSA-NCAM signal in RMS region 1 (CD) identified by blue box in (A). **(C)** Representative images of $Ankrd11^{control}$ and $Ankrd11^{nscKO}$ of P15 RMS region 1 (caudodorsal) immunostained for PSA-NCAM (blue) and Hoechst (white). Blue box indicates RMS region 1 that is shown in higher magnification to the right. Yellow dashed line indicates RMS defined by PSA-NCAM boundaries as seen in NEUN and CC3 analysis in Fig. 4. Yellow box indicates RMS R1 image exactly shown in Fig. 4N and representative location of RMS R1 image in Fig. 4K. Error bars represent SEM. Data was analyzed using unpaired t-test. n=4-5 mice per genotype from at least two independent litters, ns=not significant. Scale bars represent 500 µm (C), 250 µm (A), 100 µm (inset of A and C). Ctx=cortex; DS=dorsal striatum; RMS=rostral migratory stream; R1=region 1.
